# Synbiotic supplementation with prebiotic green banana resistant starch and probiotic *Bacillus coagulans* spores ameliorates gut inflammation in mouse model of inflammatory bowel diseases

**DOI:** 10.1007/s00394-020-02200-9

**Published:** 2020-02-17

**Authors:** Tanvi Shinde, Agampodi Promoda Perera, Ravichandra Vemuri, Shakuntla V. Gondalia, David J. Beale, Avinash V. Karpe, Sonia Shastri, Waheedha Basheer, Benjamin Southam, Rajaraman Eri, Roger Stanley

**Affiliations:** 1 Centre for Food Safety and Innovation, Tasmanian Institute of Agriculture, University of Tasmania, Launceston, TAS 7250 Australia; 2 School of Health Sciences, College of Health and Medicine, University of Tasmania, Launceston, TAS 7250 Australia; 3 Centre for Human Psychopharmacology, Swinburne University of Technology, Hawthorn, VIC Australia; 4 Health and Biosecurity, Commonwealth Scientific and Industrial Research Organization (CSIRO), Gate 13 Kintore Avenue, Adelaide, SA 5000 Australia; 5 Land and Water, Commonwealth Scientific and Industrial Research Organization (CSIRO), Ecosciences Precinct, Dutton Park, QLD 4102 Australia

**Keywords:** Synbiotic, Prebiotic, Probiotic, Inflammatory bowel diseases, *Bacillus* spores, Green banana, Resistant starch, Mucosal barrier, Short-chain fatty acids

## Abstract

**Purpose:**

The research goal is to develop dietary strategies to help address the growing incidence of inflammatory bowel diseases (IBD). This study has investigated the effectiveness of green banana resistant starch (GBRS) and probiotic *Bacillus coagulans* MTCC5856 spores for the amelioration of dextran-sulfate sodium (DSS)-induced colitis in mice.

**Methods:**

Eight-week-old C57BL/6 mice were fed standard rodent chow diet supplemented with either *B. coagulans*, GBRS or its synbiotic combination. After 7 days supplementation, colitis was induced by adding 2% DSS in drinking water for 7 days while continuing the supplemented diets. Animal health was monitored and after 14 days all animals were sacrificed to measure the biochemical and histochemical changes associated with each supplement type.

**Results:**

The disease activity index and histological damage score for DSS-control mice (6.1, 17.1, respectively) were significantly higher (*p* < 0.0001) than the healthy mice. Synbiotic supplementation alleviated these markers (− 67%, − 94% respectively) more adequately than *B. coagulans* (− 52%, − 58% respectively) or GBRS (− 57%, − 26%, respectively) alone. Compared to DSS-control synbiotic supplementation significantly (*p* < 0.0001) maintained expressions of tight junction proteins. Moreover, synbiotic effects accounted for ~ 40% suppression of IL-1β and ~ 29% increase in IL-10 levels in serum while also reducing C-reactive protein (− 37%) compared to that of the DSS-control. While, *B. coagulans* alone could not induce additional levels of short-chain fatty acid (SCFA) production beyond the caecum, the synbiotic combination with GBRS resulted in substantial increased SCFA levels across the whole length of the colon.

**Conclusion:**

The synbiotic supplementation with *B. coagulans* and GBRS ameliorated the overall inflammatory status of the experimental IBD model via synergistic functioning. This supports researching its application in mitigating inflammation in human IBD.

**Electronic supplementary material:**

The online version of this article (10.1007/s00394-020-02200-9) contains supplementary material, which is available to authorized users.

## Introduction

The full pathogenesis of inflammatory bowel diseases (IBD), including Crohn’s disease (CD) and ulcerative colitis (UC), still remains unclear. Emerging evidence substantiates the role of interactions between genetic, environmental and immunological factors. Perturbations in the composition of the gut microflora (dysbiosis) are also associated with the occurrence of IBD [1–3]. As diet is a major factor influencing the enteric microflora, numerous research projects have, therefore, considered the role of specific nutrients in the development of IBD. The Western diet, characterised by low intake of dietary fibre, has been linked with increased risk of IBD in several studies [4]. It is also implicated in leading to gut dysbiosis that further aggravates gut inflammation. In this regard, prebiotic dietary fibres and probiotics are considered as critical components of dietary improvements in the context of IBD. Both are bioactive agents that function to suppress inflammation via a number of proposed mechanisms [5–7]. Hence, various probiotic and prebiotic agents are being increasingly explored to treat or prevent IBD in humans [7–9].

A number of factors influence the beneficial effects of probiotics. Their survival in delivery formats, including functional foods, is required. They must also survive during gastric transit to exert health effects of the live organism [10]. In this context, *Bacillus* species are a growing research focus due to the ability of their heat-stable spores to survive gastric transit, harsh manufacturing and storage temperatures and delivery formats that potentially involve hot foods [11, 12]. Furthermore, *Bacillus* strains are known to exert therapeutic effects owing to their ability to induce immune responses and to produce antimicrobial peptides that help mitigate inflammation [12]. *Bacillus coagulans* MTCC 5856 spores, specifically, have been shown to survive during adverse processing and storage conditions of functional foods [13], survive gastric transit, induce significant immunomodulatory effects in vitro [14] and exhibit beneficial effects in therapeutic management of clinical diarrhea [15].

An additional factor that may influence the beneficial effects of probiotics is their ability to generate fermentation products that influence the composition of the gut microbiome. This, in turn, can potentially improve the health of the host. Bacteriocins and organic acids are two possible antimicrobial products that can be produced by probiotics to influence and stabilize the gut microbiome [16, 17]. Other intermediate metabolites, including short-chain fatty acids (SCFAs) produced as a consequence of bacterial fermentation of prebiotics in the gut, have been affirmed to exert beneficial effects on the host [6, 18]. The probiotic effect could, therefore, be potentiated by co-supplementation with prebiotic dietary fibres that can be metabolised and fermented by the administered probiotic, as well as by beneficial microflora in the gut. The resulting production of metabolites can direct the shift of immune markers from pro-inflammatory to anti-inflammatory phenotype [19]. This combination of probiotic and prebiotic factors can be referred to as synbiotic. The approach potentially offers greater success of colonisation and survivability of beneficial bacteria owing to the greater effects that can be achieved compared to using either probiotics or prebiotics alone [20]. *B. coagulans* MTCC5856 is known to ferment variety of plant fibres [21–23]. It is, therefore, a candidate probiotic for synbiotic combination with plant prebiotic fibres.

Resistant starch (RS) is a plant prebiotic classified as a type of dietary fibre [24]. RS is defined as the sum of starch and the degradation products of starch that, on average, reaches the large intestine of healthy adult humans. Numerous clinical studies have successfully demonstrated the beneficial effects of RS on colonic health [24, 25]. Dried green banana (GB), ground to a flour, is a high volume natural food source of RS. It has been demonstrated to prevent intestinal inflammation [26] and modulate oxidative stress [27] in animal models of colitis. It can also impart anti-diarrhoeal effects in children [28, 29]. GB flour is also being increasingly incorporated in food products to avoid gluten and provide food functionality such as stabilising emulsions [30–32]. Therefore, the combination of GB and *B. coagulans* could be used to create synbiotic functional foods targeted towards improving gut health. An effective combination would be of high interest for health food applications such as for mitigating inflammation in IBD patients. However improved knowledge on possible health effects, dosage response and mechanisms is needed to support such developments.

This study aimed to investigate the efficacy of pre-conditioning the gut, using a diet supplemented with *B. coagulans* spores and GBRS, on ameliorating the severity of colitis in mice. By feeding the ingredients both individually and in combination, knowledge can be gained on the extent of synergistic interactions and the underlying mechanisms that generate the health effects.

## Materials and methods

### Probiotic bacteria and prebiotic dietary fibre

LactoSpore^®^ containing probiotic strain *B. coagulans* MTCC 5856 (6 × 10^9^ spores/g) was produced by Sami Labs Limited (Bangalore, India) and supplied by Sabinsa Corporation (Australia). Prebiotic green banana resistant starch (GBRS) flour (dietary fibre content—50%) was supplied by Natural Evolution™, Australia. It is a product made by low-temperature drying of *Musa acuminata* cv Lady Finger. The nutritional composition of the GBRS is detailed in Supplementary Table 1.

### Animals

Fifty C57BL/6J (7-week old) mice of both sexes of average weight 19 ± 1 g were obtained from the University of Tasmania animal breeding facility. They were housed in a temperature-controlled environment with a 12-h day/night light cycle. Following an initial acclimation period of 7 days, the individual body weights were assessed daily during the experimental trial. All mice had ad libitum access to radiation-sterilised rodent feed pellets (Barastoc Rat and Mouse, Ridley AgProducts, Australia) and autoclaved tap water for drinking during experiments. All animal experiments were approved by the Animal Ethics Committee of the University of Tasmania (ethics approval number: A0015840) and conducted in accordance with the Australian Code of Practice for Care and Use of Animals for Scientific Purposes (8th Edition, 2013). All efforts were made to minimize animals’ suffering and to reduce the number of animals used.

### Study design and treatments

**Fig. 1:**
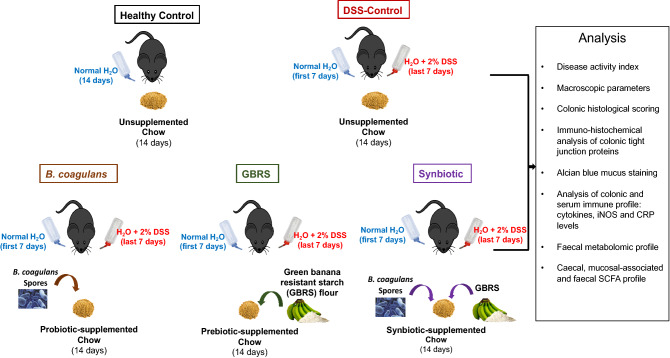
Experimental design of in vivo feeding trial to analyse prophylactic efficacy of *B. coagulans* spores, GBRS and synbiotic in DSS-induced acute colitis mice model. C57BL/6J mice (*n* = 10 per group) were fed chow supplemented with either *B. coagulans* spores, GBRS or their synbiotic combination for 14 days. Colitis was induced by administration of 2% DSS in drinking water for the last 7 days

Following 1 week of acclimation, mice at 8 weeks of age were randomly allocated into the following 5 groups (*n* = 10 per group): (1) Healthy Control (HC), (2) DSS-control, (3) Probiotic *B. coagulans* MTCC 5856 (*B. coagulans*), (4) Prebiotic green banana resistant starch (GBRS) and (5) synbiotic *B. coagulans* and GBRS combination. The experimental design of the mice feeding trial is illustrated in Fig. 1. Mice in HC and DSS-control groups received 4 g chow mash (standard rodent chow pellet blended with water to form a mash and served to mice in 30 mm petri dishes placed in the cages). The standard rodent chow contained DF content of 3.2% crude fibre and 10.4% Neutral Detergent fibre as detailed in Supplementary Table 2. The *B. coagulans* group received 4 g chow mash supplemented with probiotic *B. coagulans* MTCC 5856 spores (2 × 10^9^ CFU/day/mouse). The GBRS group received 4 g chow mash supplemented with GBRS (400 mg/day/mouse). The synbiotic group received 4 g chow each supplemented with *B. coagulans* MTCC 5856 spores (2 × 10^9^ CFU/day/mouse) and GBRS (400 mg/day/mouse). The chow mash was prepared fresh each day. The mice were single-caged throughout the experiment to measure the defined daily intake of respective treatments from prepared chow mash. The mice were fed these treatments for 14 days. Colitis was induced during the last 7 days of the experimental period by administering 2% dextran sulfate sodium (DSS; MP Biomedicals, colitis grade average molecular weight: 36,000–50,000) in the drinking water of all groups except for non-colitic control mice which received normal drinking water. Mice were sacrificed on day 15 by CO_2_ asphyxiation.

### Clinical scoring and histological analysis

A disease activity index (DAI) was determined daily in all mice by scoring for body weight, hemoccult reactivity, presence of gross blood and stool consistency during the week of DSS induction. DAI was determined by combining the scores from these three categories as described previously [19]. Faecal samples were collected on day 14 and stored at − 80 °C for metabolite analysis. After sacrificing the mice, the colons were dissected from the caecum to the anus as described previously [19]. Spleen weight, colon length, colon weight/body weight ratio were calculated as macroscopic markers of inflammation. The mucosal and caecal contents were collected by scraping with sterile pipette tips for SCFA and metabolite profiling and stored at − 80 °C. The colon was bisected longitudinally, and one half was prepared using the Swiss roll technique [33], whereas the remaining colonic tissue was dissected out, segregated into proximal colon (PC) and distal colon (DC) and snap-frozen for molecular analyses. Swiss rolls underwent 24 h fixation in 10% (v/v) neutral-buffered formalin. Swiss rolls were subsequently transferred to 70% ethanol prior to progressive dehydration, clearing and infiltration with HistoPrep paraffin wax (Fisher Scientific, Philadelphia, PA, USA). Swiss rolls were then embedded in wax and 5 μm sections were cut using a rotary microtome. For histological analysis, proximal and distal colon tissue sections (*n* = 8 per group) were stained with haematoxylin and eosin stain (H and E; HD Scientific, Sydney, Australia). The slides were graded by assessors blinded to the experimental treatments for the severity of tissue damage at distal and proximal regions as described previously [19]. All images were captured on a Leica DM500 microscope using a Leica ICC50 W camera (Leica Microsystems, Wetzlar, Germany).

### Alcian blue staining

DSS-induced alterations in goblet cells, and subsequent depletion in synthesis and secretion of mucin glycoprotein, were analysed by Alcian blue staining (ab150662 Alcian Blue, pH 2.5 (Mucin Stain), Abcam, Australia) following the manufacturer’s instructions. The staining intensity (IOD) was assessed using Image Pro Plus 7.0 (Media Cybernetics, Inc., Rockville, MD, USA) and used for comparison among groups [34].

### Immunohistochemical detection of tight junction proteins

Immunohistochemical detection of epithelial tight junction (TJ) proteins: ZO-1, occludin and claudin-1 was performed using a Rabbit specific HRP/DAB (ABC) Detection IHC kit (ab64261, Abcam, Australia) following the manufacturer’s instructions and as previously described [19]. Antibodies anti-ZO-1 (NBP1-85046, Novus Biologicals, Australia, 1:400); anti-occludin (NBP1-87402, Novus, 1:600) and anti-claudin-1 (NBP1-77036, Novus, 1 μg/mL) were used for incubating the colonic sections overnight at 4 °C. Computer-assisted image analysis was performed with a Leica DM500 microscope (Leica Microsystems, Wetzlar, Germany), Leica ICC50 W camera (Leica Microsystems, Wetzlar, Germany), and Image Pro Plus 7.0 (Media Cybernetics, Inc., Rockville, MD, USA) software. The expression of tight junction (TJ) proteins: ZO-1, occludin and claudin-1 was blindly assessed by choosing random five fields on each slide (*n* = 4/group). Barrier TJ protein expressions and staining intensity in colonic epithelium were expressed as the percentage expression of a respective TJ protein.

### Myeloperoxidase activity

The extent of the inflammatory cell invasion in the colon was examined by the assessment of myeloperoxidase (MPO) activity [35]. Weighed and snap-frozen PC and DC specimens (*n* = 3) were analysed for MPO activity using a Myeloperoxidase Activity Assay kit (ab105136, colorimetric, Abcam^®^, Cambridge, UK) following the manufacturer’s instructions. The values are expressed as MPO activity units/g tissue.

### Tissue explant culture and cytokine measurements

PC (*n* = 3) and DC (*n* = 3) colon tissues of mice from each group were cut, weighed and washed with cold phosphate-buffered saline (PBS) before transferring to a 12-well plate containing 0.5 mL/well of RPMI1640 culture medium (In Vitro Technologies Pty Ltd, Melbourne, Australia) supplemented with 10% v/v foetal calf serum (Gibco, Life Technologies Pty Ltd, Melbourne, Australia), penicillin (100 mU/L), and streptomycin (100 mg/L) (Sigma-Aldrich Pty Ltd, Sydney, Australia) as described previously [36]. After 24 h of incubation, the supernatant was collected from each well, centrifuged and stored at − 80 °C until further analysis. Serum was collected from blood drawn by cardiac puncture at the end of the study for cytokine analysis.

The cytokine levels in colon tissue (*n* = 3) and serum (*n* = 3) were determined by immunoassay using a Bio-Plex Pro Mouse cytokine 23-plex kit (Bio-Rad #M60009RDPD, Bio-Rad Laboratories, Gladesville, NSW, Australia) following the manufacturer’s instructions and concentrations analysed using a Bio-Plex 200 instrument (Bio-Rad) and Bioplex Manager software, version 6 (Bio-Rad Laboratories) respectively [36]. For tissues, the cytokine levels were normalized by dividing the cytokine results (pg/mL) by the measured biopsy weight (g). The most significantly altered cytokines are presented as pg/g of tissue.

### iNOS activity

The expression of inducible isoforms of nitric oxide synthase (iNOS) in colonic epithelial cells in response to pro-inflammatory stimuli [37] was determined in PC (*n* = 3) and DC (*n* = 3) specimens using a Nitric Oxide Synthase Activity Assay kit (ab211084, Fluorometric, Abcam^®^, Cambridge, UK), following the previously described method [19]. The results are expressed as iNOS activity mU/mg.

### Serum C-reactive protein analysis

The levels of C-reactive protein (CRP) in serum from respective groups (*n* = 3 samples/group) were analysed using Mouse C-Reactive Protein/CRP Quantikine Elisa kit (MCRP00, R&D Systems, Australia) following the manufacturer’s instructions. The results are expressed as μg/mL.

### Volatile SCFA analysis

The contents of colon (mucosal-associated) and caecum were collected by scraping with sterile pipette tips for SCFA profiling and stored at − 80 °C. GC–MS analysis of 100–150 mg fresh weight (stored at − 80 °C) of caecal, mucosal-associated and faecal samples (*n* = 5 per group) each was used for volatile SCFA profiling following the method described previously [19, 38]. The samples were prepared and derivatized following the protocol developed by Furuhashi et al. [39] with some modifications. Briefly, caecal, mucosal-associated and faecal samples of 100–150 mg fresh weight (stored at − 80 °C) were weighed to ± 0.1 mg accuracy. These samples were added to a sterile 1.5 mL bead-beating tube (NAVY Rino Lysis tubes, Next Advance, Troy, NY, USA). A 1.0 mL aliquot isobutanol (10% MilliQ water), (LC–MS grade, Merck, Castle Hill, NSW, Australia) was added to each sample, followed by two 30 s, 4000 rpm homogenization pulses sandwiched between a 20-s pause interval (Precellys Evolution Homogenizer, Bertin Instruments, Montigny-le-Bretonneux, France). The samples were subsequently centrifuged at 16,000×*g* for 6 min.

The supernatant (675 µL) was transferred to a clean round-bottomed 2 mL centrifuge tube (Eppendorf South Pacific Pty. Ltd., Macquarie Park, NSW, Australia) and NaOH (20 mM, 125 µL, Merck Pty Ltd., Castle Hill, NSW, Australia) and chloroform (400 µL, LC–MS grade, Merck Pty Ltd.,) were added. The samples were briefly vortexed and centrifuged at 16,000*g* for 3 min. The aqueous phase (upper layer, 400 µL) was transferred to a new clean round-bottomed 2 mL centrifuge tube (Eppendorf South Pacific Pty. Ltd., Macquarie Park, NSW, Australia) containing a boiling chip (Sigma Aldrich, Castle Hill, NSW, Australia). Pyridine (100 µL), isobutanol (80 µL) (both LC–MS grade, Sigma Aldrich, Castle Hill, NSW, Australia), and MilliQ (Millipore Corporation) water (70 µL) were added and the samples were subjected to gentle hand vortexing (swirling action) followed by the addition of 50 µL isobutyl chloroformate (98% purity, Sigma Aldrich, Castle Hill, NSW, Australia). The tube was kept opened to release any generated gases and was allowed to stand for about 1 min. Hexane (150 µL, LC–MS grade, Sigma Aldrich, Castle Hill, NSW, Australia) was then added to each tube, which was then capped and vortexed prior to centrifugation at 15,700×*g* for 4 min. The upper phase (100 µL) was subsequently transferred to clean gas chromatography (GC) autosampler vial fitted with silanized low volume glass inserts; Malathion (1 µL, equivalent to 2.5 µg dry weight) was added as an internal standard.

The GC–MS analysis was performed on an Agilent 6890B GC oven coupled to a 5977B mass spectrometer (MS) detector (Agilent Technologies, Mulgrave, VIC, Australia) fitted with an MPS autosampler (Gerstel GmbH & Co.KG, Mülheim an der Ruhr, Germany). The GC oven was fitted with two 15 m HP‐5MS columns (0.25 mm ID and 0.25 µm film thickness; 19091S-431 UI (Ultra Inert), Agilent Technologies, VIC, Australia) coupled to each other through a purged ultimate union (PUU) for the use of post-run backflushing. The sample (1.0 µL) was introduced via a multimode inlet (MMI) operated in split mode (1:20). The column was maintained at 40 °C for 5 min, followed by an increase to 250 °C at a rate of 10 °C/min. This was followed by a second increment to 310 °C at a rate of 60 °C/min. The column was held at 310 °C for 1 min. The mass spectrometer was kept in extractor ion mode (EI mode) at 70 eV. The GC–MS ion source temperature and transfer line were kept at 250 °C and 280 °C, respectively. Detector voltage was kept at 1054 V. The MS detector was turned off for the first 3 min and, at 4.0–4.8 min and 12.5- to 13.2-min time windows until the excess derivatization reagent (chloroformate/hexane solvents) were eluted from the column. This ensured that the source filament was not saturated and damaged. The scan range was kept in the range of *m/z* 35–350 (35–350 Da). Data acquisition and spectral analysis were performed as described in a previous study [38] and qualitative identification of metabolites was performed according to the Metabolomics Standard Initiative (MSI) chemical analysis workgroup [40] using standard GC–MS reference metabolite libraries (NIST 17, and an in-house CF-based metabolomics library developed after Smart et al. [41] with the use of Kovats retention indices based on a reference *n*-alkane standard (C8-C40 Alkanes Calibration Standard, Sigma-Aldrich, Castle Hill, NSW, Australia).

### Metabolomic analysis

In addition, untargeted GC–MS metabolomic profiling of faecal samples (*n* = 5 per group) was performed using GC–MS analysis as described previously [19, 38]. The samples were subjected to derivatisation to increase volatility before subjecting to GC–MS analysis. Briefly, the samples (*n* = 5, weight = 40 mg) were freeze-dried and suspended in 1 mL methanol (LC–MS grade, Merck, Castle Hill, NSW, Australia), supplemented with 10 µg/mL adonitol (Analytical grade, Sigma Aldrich, Castle Hill, NSW, Australia) as an internal standard in a sterile 2 mL bead-beating tube. The samples were homogenized by bead beating for 30 s and then centrifuged at 570 g/4 °C for 15 min. The supernatant (50 µL) was transferred to a fresh centrifuge tube (1.5 mL) and dried in a vacuum evaporator centrifuge (LabGear, Brisbane, QLD, Australia) at 35 °C. Methoxyamine-HCl (20 mg/mL in Pyridine) (both, Analytical grade, Sigma Aldrich, Castle Hill, NSW, Australia) was added (40 µL) and samples were incubated at 30 °C/1400 rpm (ThermoMixer C, Eppendorf, Hamburg, Germany) for 90 min. This was followed by sialylation with 70 µL BSTFA at 37 °C/1400 rpm for 30 min. Pre-derivatized 13C-stearic acid (10 µg/mL) was added (1 µL) as the QA/QC internal standard. The mixture was briefly vortexed and centrifuged at 15,700*g* for 5 min. The aliquot was transferred to vials for GC–MS analysis.

The GC–MS analysis was performed on an Agilent 6890B gas chromatograph (GC) oven coupled to a 5977B mass spectrometer (MS) detector (Agilent Technologies, Mulgrave, VIC, Australia) fitted with an MPS autosampler (Gerstel GmbH & Co. KG, Deutschland, Germany). The GC–MS conditions were as stated previously [42–44]. Data acquisition and spectral analysis were performed using the Qualitative Analysis software (Version B.08.00) of MassHunter Workstation (Agilent Technologies). Qualitative identification of the compounds was performed according to the Metabolomics Standard Initiative (MSI) chemical analysis workgroup [40] using standard GC–MS reference metabolite libraries (NIST 17, Fiehn Metabolomics RTL Library (G166766A, Agilent Technologies) and the Golm database) and with the use of Kovats retention indices based on a reference *n*-alkane standard (C8-C40 Alkanes Calibration Standard, Sigma-Aldrich, Castle Hill, NSW, Australia). For peak integration, a five-point detection filtering (default settings) was set with a start threshold of 0.2 and a stop threshold of 0.0 for 10 scans per sample. Procedural blanks (*n* = 7) were analysed randomly throughout the sequence batch. The obtained data were processed on the Quantitative Analysis software of MassHunter Workstation and exported as a Microsoft Excel output file for statistical analysis.

GC–MS data imported to Microsoft Excel platform was normalized with respect to the internal standard adonitol (relative standard deviation = 11.257%). The normalized data was further log-transformed and auto-scaled (mean-centred) before statistical analysis [45]. For analysis of metabolome variations, partial least squares-discriminant analysis (PLS-DA) and orthogonal (O) PLS-DA were used. Because PLS-DA can overfit data, we used 1000 permutations to validate these models. The OPLS-DA was used to identify discrimination between metabolites contributing to classification as previously described [38].

### Statistical analysis

The samples in the study were randomly chosen for all the analyses to avoid bias. All data are presented as means ± standard error of the mean (SEM). The statistical analysis was performed with the use of GraphPad Prism Software (Version 7.0, San Diego, CA, USA). The data were evaluated using One-way analysis of variance (ANOVA) followed by Tukey’s post hoc test to determine statistical differences between the groups against the DSS-control samples. For the analysis of DAI and body weight changes during the experimental period, two-way ANOVA followed by Tukey’s post hoc test was used, setting treatment and the time as the variables. A *p* value of < 0.05 was considered significant. A MetaboAnalyst (Version 4.0) data annotation approach and Kyoto Encyclopaedia of Genes and Genomes (KEGG) Pathway Database were used for the hierarchical clustering analysis and significance analysis for microarrays (SAM), along with the variable importance of projection (VIP) [46]. The SAM and VIP methods are well-established statistical methods for metabolites and were used to select the most discriminant and interesting biomarkers [47].

## Results

### Effects of *B. coagulans*, GBRS and synbiotic supplementation on clinical manifestations and macroscopic inflammatory markers

**Fig. 2:**
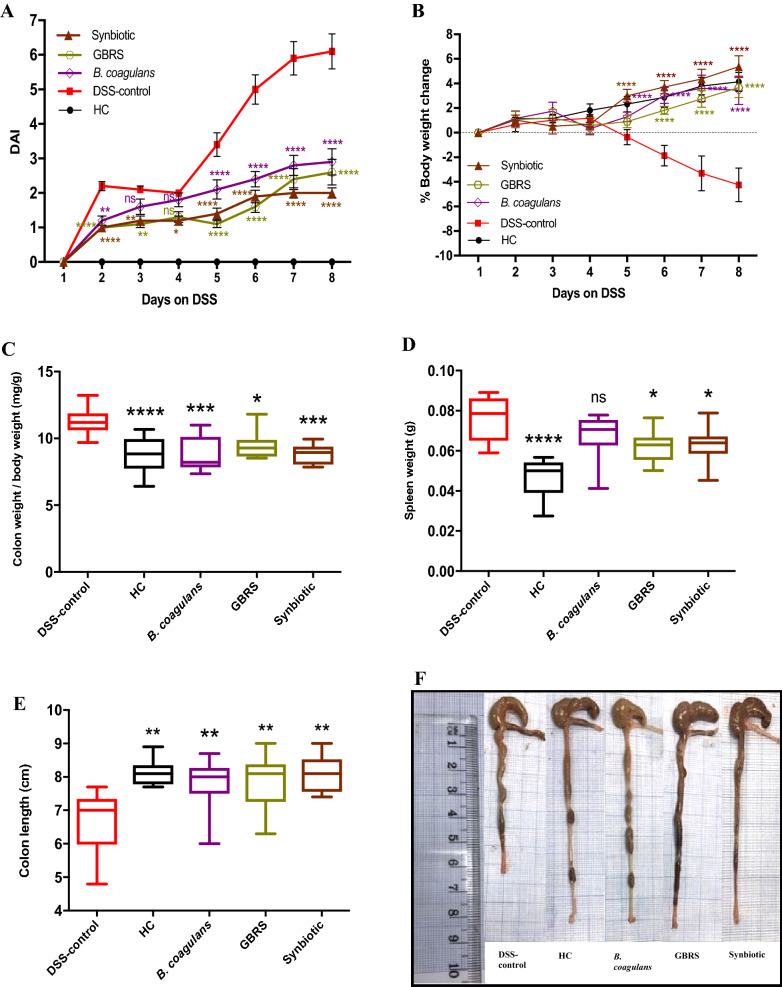
Effect of *B. coagulans* spores, GBRS and synbiotic on clinical manifestations in DSS-induced colitis mice. **a** Disease activity index (DAI), **b** % body weight change. Statistical significance among groups evaluated by two-way repeated-measures analysis of variance (ANOVA) followed by Tukey’s test. *p < 0.05, **p < 0.01, ***p < 0.001, ****p < 0.0001 vs. DSS-control group and data expressed as mean ± SEM (*n* = 10 per group). Colon weight/body weight ratio (**c**), spleen weight (**d**), colon length (**e**) and macroscopic appearance of colon (**f**). Data expressed as mean ± SEM (*n* = 10 per group), evaluated by one-way ANOVA followed by Tukey’s test. *NS* non-significant

DSS-induction resulted in a progressive increase in colonic inflammation as demonstrated by severe body weight loss and high DAI (Fig. 2a, b). However, supplementation of *B. coagulans*, GBRS and synbiotic treatments started 7 days prior to DSS-induction, attenuated the impact of the DSS damage and boosted the recovery of the treated animals. This is evidenced by the significant reduction in body weight loss and by lower incidences of diarrheic/bloody faeces, resulting in lower DAI values throughout the experiment in the treated groups when compared to the untreated DSS-control group. At the end of the experiment on day 8, DAI was significantly (*p* < 0.0001) higher for the DSS-control (6.1 ± 0.5) compared to *B. coagulans* (2.9 ± 0.4, 52% reduction), GBRS (2.6 ± 0.4, 57% reduction) and synbiotic (2 ± 0.2, 67% reduction) mice (Fig. 2b). Moreover, probiotic, prebiotic and synbiotic supplementation significantly (*p* < 0.0001) reduced the loss of body weight compared to that of DSS-control group starting from day 6.

The macroscopic evaluation of colonic segments affirmed the remedial effects of all three treatments used in our study. This was indicated by a substantial reduction in colon weight/body weight ratio (*B. coagulans*, 7.70 ± 0.2; GBRS, 9.32 ± 0.3 and synbiotic, 8.3 2 ± 0.3 mg/g) compared with the DSS-control group (11.16 ± 0.2 mg/g) (Fig. 2c). The relative spleen weights of the DSS-control mice were also markedly higher (0.08 ± 0.004 g) than that of GBRS (0.063 ± 0.002 g) and synbiotic (0.062 ± 0.003 g) mice (Fig. 2d). *B. coagulans* had no effect on spleen weight reduction (0.068 ± 0.004 g). *B. coagulans* (7.80 ± 0.3 cm), GBRS (7.91 ± 0.2 cm) and synbiotic (8.09 ± 0.2 cm) supplementation effectively prevented the colon shortening compared with the DSS-control group (6.80 ± 0.3 cm) (Fig. 2e, f).

### Effects of *B. coagulans*, GBRS and synbiotic supplementation on histological alterations in colon

**Fig. 3:**
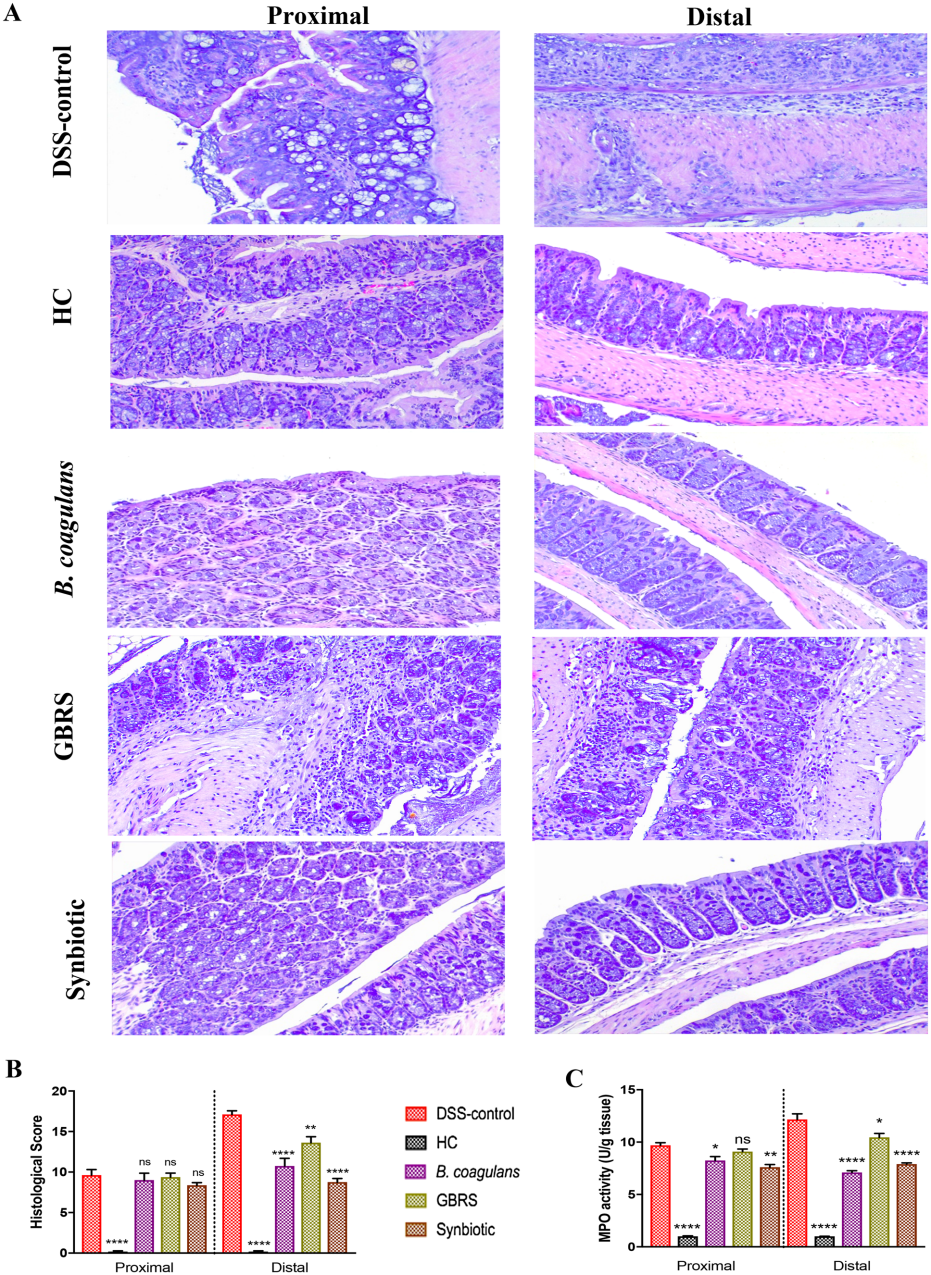
Effect of *B. coagulans* spores, GBRS and synbiotic treatments on DSS-induced colonic tissue injury. **a** Histological images of proximal and distal colonic tissues stained with hematoxylin and eosin at × 20 for each experimental group. **b** Histological score calculated after microscopic analyses of proximal and distal sections of the colon. **c** Myeloperoxidase (MPO) activity in colonic tissues was determined by colorimetric assay. Results, expressed as mean ± SEM (*n* = 8 per group), were evaluated by one-way ANOVA followed by Tukey’s test (*p < 0.05, **p < 0.01, ****p < 0.0001)

Histological (H&E staining) examination of proximal colon (PC) and distal colon (DC) sections of DSS-induced mice displayed histological damage with erosion or destruction of epithelium, crypt distortion, depletion of goblet cells, submucosal oedema and inflammatory cellular infiltration in the colon, mostly affecting the distal section (Fig. 3a). While HC showed no signs of histological colon damage (score 0), DSS resulted in a cumulative damage score of 9.38 ± 0.8 for PC and 17.1 ± 0.4 for DC (Fig. 3b). Supplementation with synbiotic and *B. coagulans* induced protection against the damage, as evidenced by substantial retention of colonic structure, protection of crypts and goblet cells, and reduced infiltration of inflammatory cells. This resulted in a significant overall reduction of cumulative histological scores of DC (8.8 ± 0.5, 10.8 ± 1.0 for synbiotic and *B. coagulans* respectively). GBRS provided a partial, but significant, protection with a histological score of 13.6 ± 0.7. In contrast, histology scores for PC demonstrated no statistically significant protection by the three treatments. MPO assay, however, showed a substantial reduction in neutrophil infiltration in PC by synbiotic and *B. coagulans* compared with that of the DSS-colitic group. In DC, all three supplementations were successful in reducing the inflammatory cell infiltrate, as determined by decreased MPO activity (Fig. 3c), with *B. coagulans* and synbiotic being more effective than GBRS.

### Effects of *B. coagulans*, GBRS and synbiotic supplementation on goblet cells and colonic tight junction barrier

**Fig. 4:**
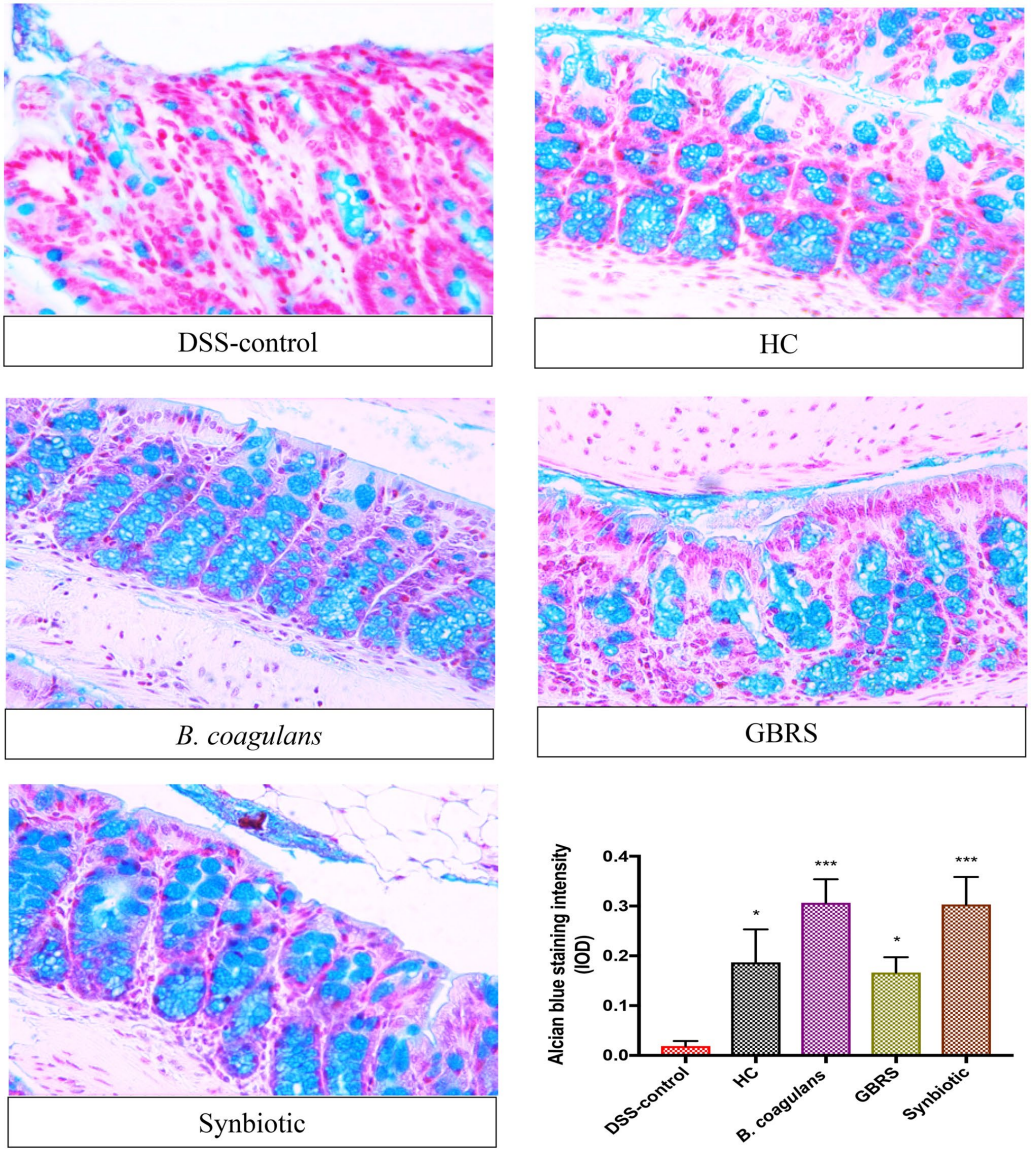
Effect of *B. coagulans* spores, GBRS and synbiotic on goblet cells. The paraffin embedded sections were stained with Alcian Blue to detect changes in goblet cells and in production of mucus in distal colonic tissue in each experimental group (× 40). Staining intensity (IOD) of respective group is illustrated in the graph. Results expressed as mean ± SEM (*n* = 4 per group), evaluated by one-way ANOVA followed by Tukey’s test (*p < 0.05, ***p < 0.001)

Staining with Alcian blue was performed to examine the effect of supplementation on DSS-induced alterations in the mucus secretion by goblet cells. Significantly high mucus staining with Alcian blue was detected in colon sections of mice supplemented with *B. coagulans* and synbiotic, with moderate staining of GBRS mucus suggesting induction of high secretion levels of mucus in DSS-challenged mice that received supplementations (Fig. 4). In comparison, in DSS-control colon sections, the goblet cells were almost entirely destroyed.

**Fig. 5:**
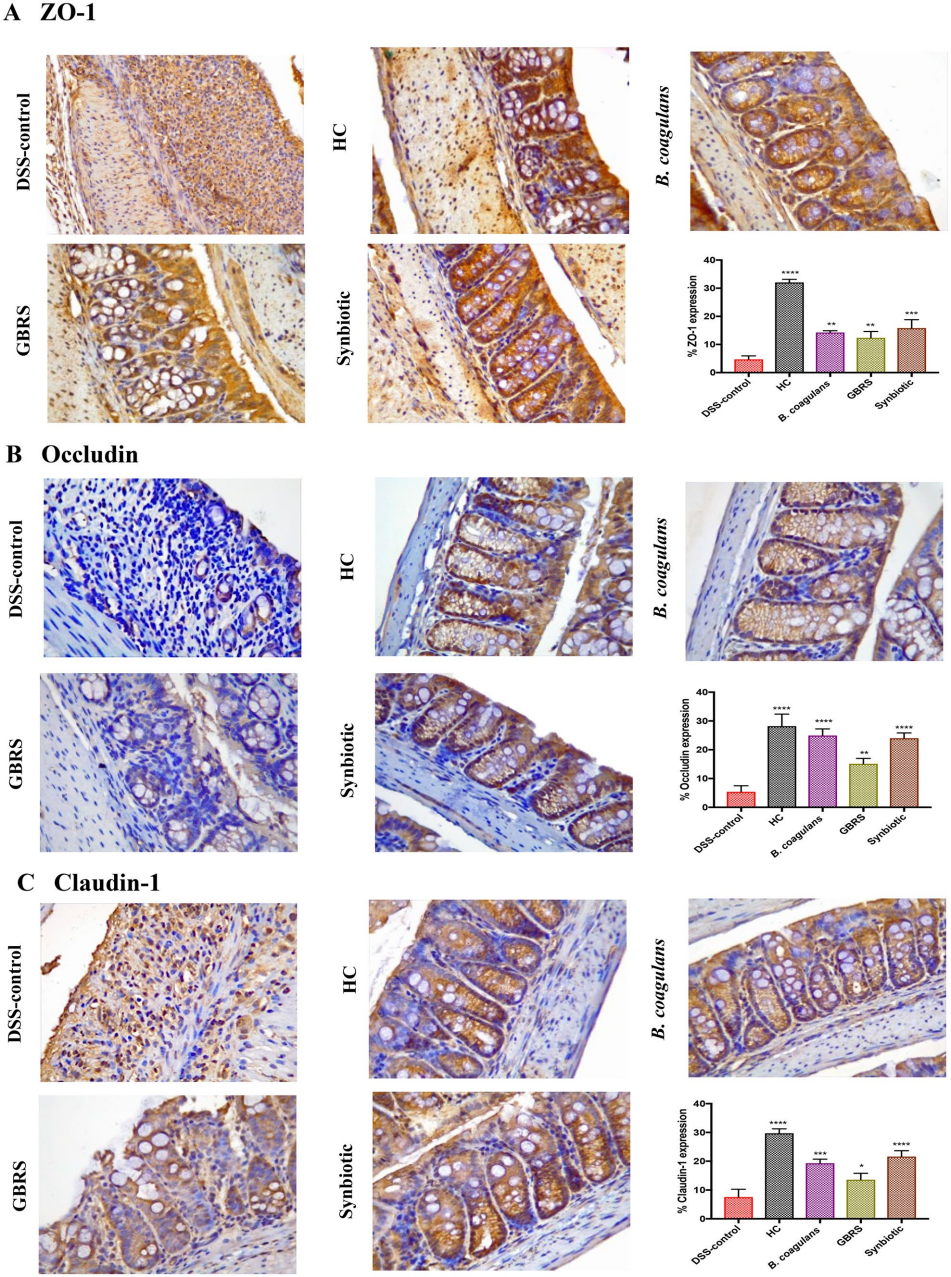
Effects of *B. coagulans* spores, GBRS and synbiotic on expression of epithelial tight junction proteins. Immunohistochemical detection of **a** ZO-1, **b** occludin and **c** claudin-1 and its respective percentage of expression in colon at × 40. Data expressed as mean ± SEM (*n* = 4 per group) and statistical significance among groups evaluated by one-way ANOVA followed by Tukey’s test *p < 0.05, **p < 0.01, ***p < 0.001, ****p < 0.0001 vs. DSS-control group

Immunohistochemical analysis was performed to investigate the impact of supplementation on the assembly of the TJs and the integrity of the intestinal barrier. The presence of the TJ proteins-ZO-1, occludin and claudin-1 were analysed for in the colonic tissue sections (Fig. 5). In HC sections, ZO-1 staining (Fig. 5a) was more intense in the apical tight junction complex, both at the surface and in the crypts. Occludin (Fig. 5b) and claudin-1 (Fig. 5c) proteins stained more strongly at the basolateral membrane of the crypts, and also showed their presence at the crypt surface. In DSS-control sections however, such signals were weak or totally absent, in line with previous reports [48, 49], indicating a low percentage of TJ protein expression. *B. coagulans* and synbiotic supplementation, however, effectively maintained the basolateral and partial apical staining of ZO-1, occludin and claudin-1 in DSS-induced mice. GBRS only displayed partial maintenance of ZO-1 staining, although the effect was less noticeable for occludin and claudin-1. In contrast, synbiotic supplementation significantly maintained the TJ patterns similar to that of HC sections, indicating a high level of protection of the integrity of the epithelium.

### Immunomodulatory effects of *B. coagulans*, GBRS and synbiotic supplementation on immune markers

**Fig. 6:**
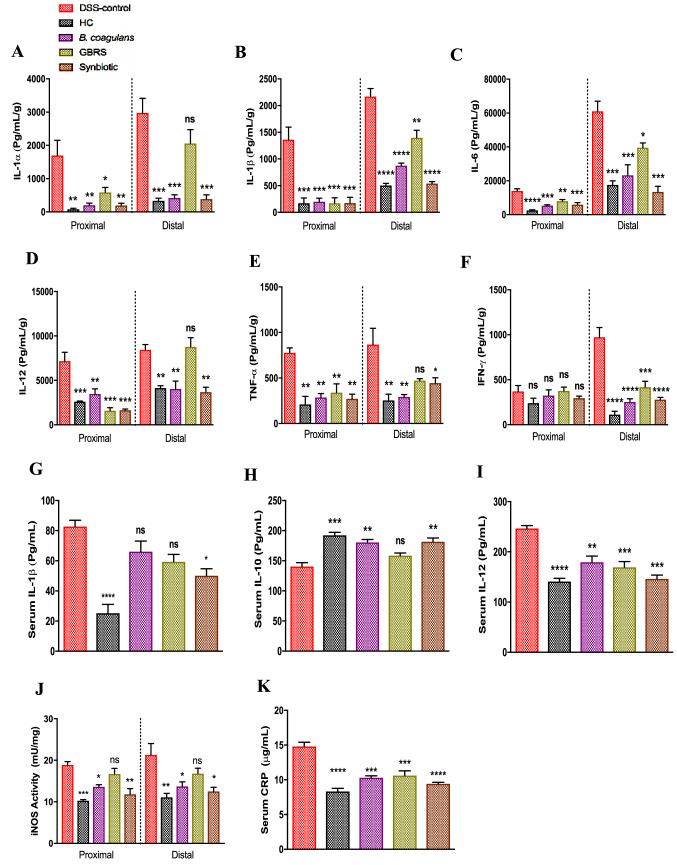
Effect of *B. coagulans* spores, GBRS and synbiotic on immune markers in colon tissues and blood serum. Protein levels of cytokines including **a** IL-1α, **b** IL-1β, **c** IL-6, **d** IL-12, **e** TNF-α, **f** IFN-γ in proximal and distal colon explants as well as cytokine levels of **g** IL-1β, **h** IL-10, and **i** IL-12 in blood serum were analysed by Bio-plex. iNOS activity in colon tissues **j** measured by NOS activity assay and CRP levels in serum **k** by ELISA. Statistical significance among groups evaluated by one-way ANOVA followed by Tukey’s test. *p < 0.05, **p < 0.01, ***p < 0.001, ****p < 0.0001 vs. DSS-control group and data expressed as mean ± SEM (*n* = 3 per group)

*Bacillus coagulans*, GBRS and synbiotic supplementation improved the altered immune responses induced by DSS thus showing their immunomodulatory and anti-inflammatory effects (Fig. 6). In comparison with the DSS-control group, all three treatments substantially reduced the tested pro-inflammatory cytokine levels of IL-1α, IL-1β, IL-6, IL-12, TNF-α, IFN-γ in PC and DC segments. However, no significant effect of supplementations was noted on levels of the other cytokines (Supplementary Fig. 1). Supplementation with *B. coagulans* alone and with synbiotic proved effective in reducing the levels of all the cytokines tested in comparison with that of the DSS-control mice. Relative to that of DSS-control, there was a pronounced reduction in increases of IL-1β (-60%), IL-6 (-62%), TNF-α (-66%) and IFN-γ (-73%) levels by *B. coagulans* supplementation in comparison with that of GBRS (IL-1β: − 36%, IL-6: − 35%, TNF-α: − 46%, IFN-γ: − 57%). However, GBRS had no significant effect on the levels of IL-1α, IL-12 and TNF-α in DC. The synbiotic supplementation proved effective in reducing the levels of all pro-inflammatory cytokines, in comparison with elevated cytokine levels in the DSS-control. Relative to that of DSS-control, the synbiotic supplementation compared with those of the GBRS supplementation also displayed greater reduction in the levels of IL-6 (− 78%, *p* = 0.012), IL-12 (− 56%, *p* = 0.003) and IFN-γ (− 71%, *p* = 0.526) as well as greater suppression in the level of IL-1β (− 75%, *p* = 0.181) compared to that of *B. coagulans*.

Serum cytokines indicative of immunomodulatory effects also followed a similar trend (Fig. 6g–i). Synbiotic significantly decreased the pro-inflammatory serum cytokine levels of IL-1β (50.1 ± 4.6 pg/mL) and IL-12 (146.4 ± 7.4 pg/mL) while, concomitantly increasing anti-inflammatory IL-10 (181.7 ± 6.1 pg/mL) levels compared with the DSS-control group (IL-1β: 82.8 ± 4.1 pg/mL, IL-12: 246.6 ± 6.0 pg/mL and IL-10: 140.5 ± 6.5 pg/mL). While, *B. coagulans* and GBRS supplementations alone were not effective in reducing increased serum IL-1β (66.1 ± 7.0 and 59.38 ± 5.0 pg/mL, respectively), substantial reduction in IL-12 (179.3 ± 12.3 and 169.5 ± 11.1 pg/mL, respectively) was achieved relative to that of the DSS-control. No statistically significant effect was observed for other serum cytokines (Supplementary Fig. 1). DSS-induction elevated iNOS activity in both PC and DC in response to the pro-inflammatory stimulus, in line with the previous report [37]. Synbiotic and *B. coagulans* lowered the iNOS activity significantly, while GBRS had no effect. Moreover, compared to the high CRP levels in DSS-control (14.81 ± 0.6 μg/mL), *B. coagulans* (10.31 ± 0.3 μg/mL), GBRS (10.61 ± 0.7 μg/mL) and synbiotic (9.4 ± 0.2 μg/mL) all reduced the serum CRP levels. Synbiotic and *B. coagulans* supplementations induced normalisation of CRP levels and were statistically similar to that of HC levels (8.3 ± 0.5 μg/mL). These observations indicated that a combination of probiotic spore and prebiotic GBRS resulted in an effective level of immunomodulatory activity.

### Effects of *B. coagulans*, GBRS, and synbiotic supplementation on alteration of faecal metabolic profile

**Fig. 7:**
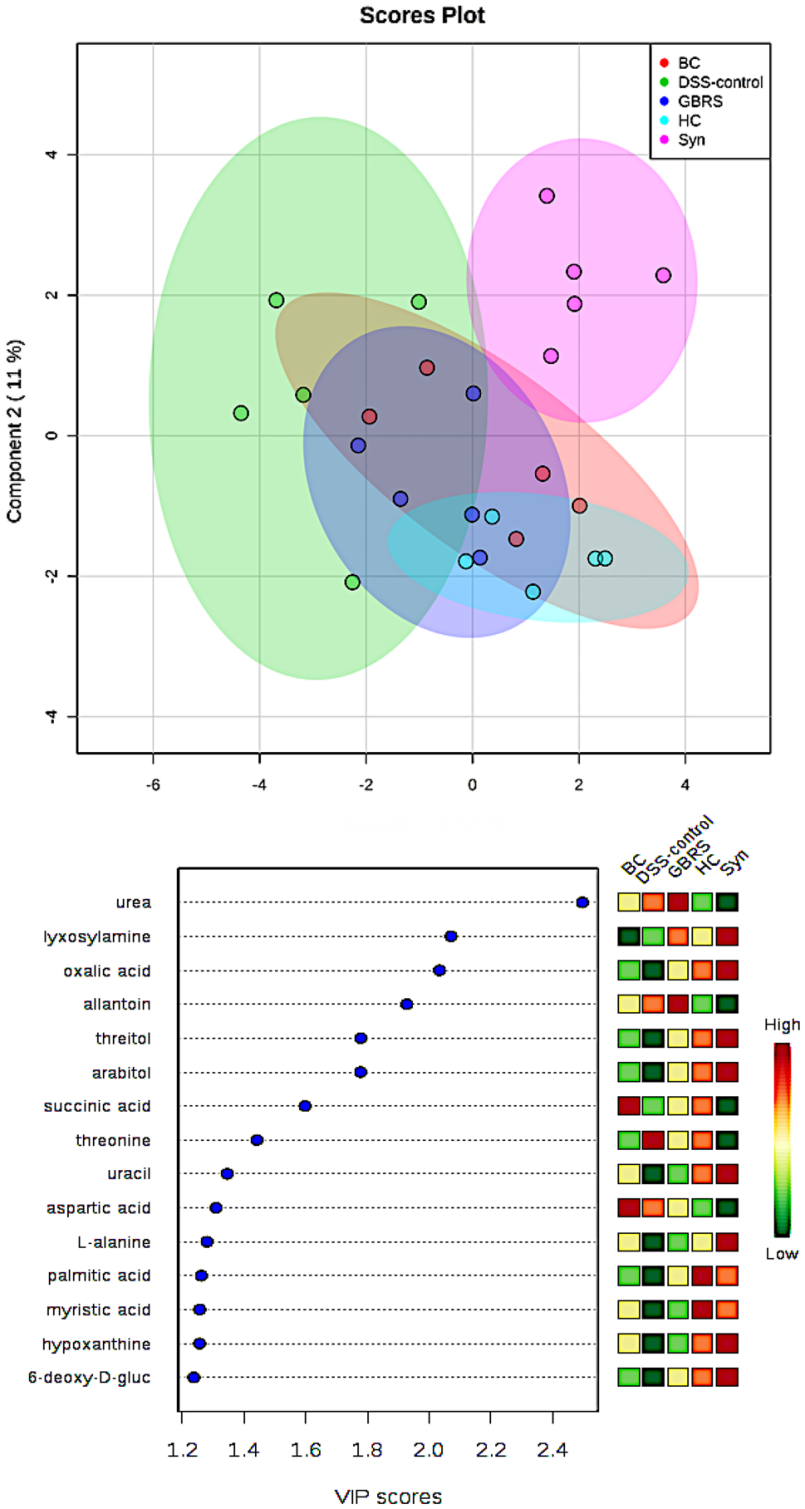
Effect of *B. coagulans* spores, GBRS and synbiotic on metabolic modulations in DSS-induced colitic mice. **a** 2D-PLS-DA plot showing spatial division among groups that received different supplementations, DSS-control mice that received no supplementation and HC. **b** Key compounds separating the groups are ranked based on variable importance projection (VIP) score plot from PLS-DA analysis. *BC* *B. coagulans*, *Syn* synbiotic

Faecal samples were analysed by GC–MS platform to gain an untargeted overview of alterations in dominant gut metabolites induced by *B. coagulans*, GBRS and synbiotic supplementations in DSS-treated mice. The analysis detected a total of 61 metabolites belonging to different functional groups such as sugars, amino acids, volatile fatty acids and biogenic amines. A supervised partial least squares-discriminant analysis (PLS-DA) was performed to evaluate metabolic phenotyping of each experimental group (Fig. 7a). The remoteness between the samples from HC and DSS-control indicates a clear distinction in metabolic patterns between the groups. Among the supplemented groups, samples from *B. coagulans* and GBRS clusters overlapped with each other, and with that of HC, and partially with DSS-control. The synbiotic cluster showed a clear divergence relative to that of the DSS-control samples suggesting its potential to induce marked changes in the metabolic profile. The combination of PLS-DA (*R*^2^*Y* = 0.803 (*p* = 0.01), *Q*^2^ = 0.521), VIP scores (Fig. 7b) and SAM enabled the identification of potential biomarkers. The results showed 61 metabolites, with 28 statistically significant compounds contributing to the clustering. Their significance analysis for microarrays (SAM) scores fold changes and International Chemical Identifiers (InChI) and standard InChI hashes (InChIKey IDs) listed in Supplementary Table 3. Key metabolic markers making a significant contribution were identified by VIP analysis as displayed in Fig. 7b. Among these identified metabolites, substantial differences in the patterns between DSS-control and HC were particularly noted for allantoin, threitol, arabitol, uracil, aspartic acid, palmitic acid, myristic acid, hypoxanthine and 6-deoxy-d-glucose. Synbiotic supplementation generated a reduction in the metabolic alterations induced by DSS (Fig. 7b and Supplementary Table 3).

### Effects of *B. coagulans*, GBRS and synbiotic supplementation on SCFA levels

**Fig. 8:**
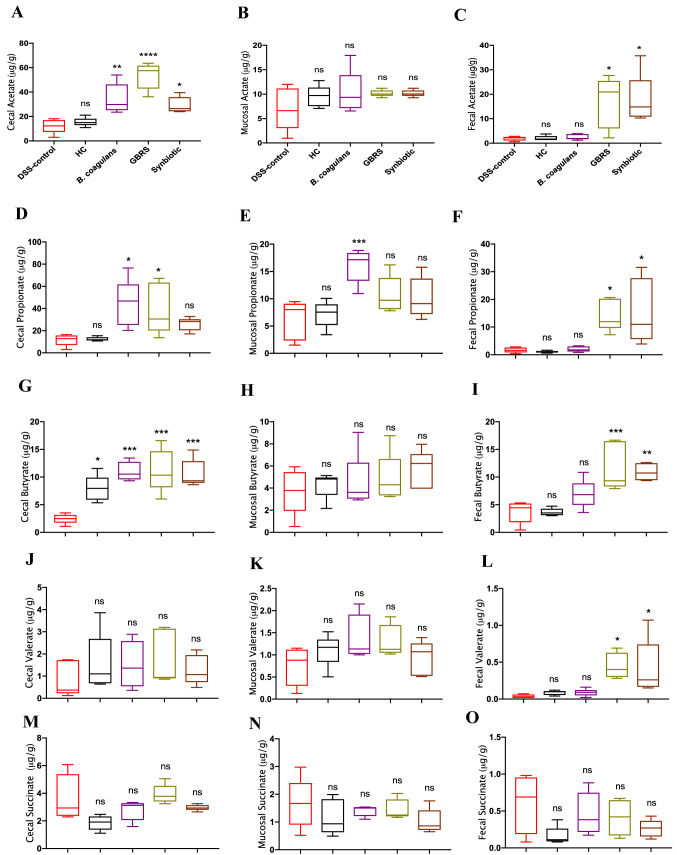
Effects of *B. coagulans* spores, GBRS and synbiotic in modulating SCFA concentrations in caecal, mucosal-associated and faecal contents in DSS-induced colitis. Caecal-acetate (**a**), propionate (**d**), butyrate (**g**), valerate (**j**), succinate (**m**); mucosal-associated acetate (**b**), propionate (**e**), butyrate (**h**), valerate (**k**), succinate (**n**) and faecal-acetate (**c**), propionate (**f**), butyrate (**i**), valerate (**l**), succinate (**o**). Statistical significance among groups evaluated by one-way ANOVA followed by Tukey’s test. *p < 0.05, **p < 0.01, ***p < 0.001, ****p < 0.0001 vs. DSS-control group and data expressed as mean ± SEM (*n* = 5 per group). *ns* non-significant

DSS-induction lead to variation in SCFA production compared to that of HC across the samples of intestinal contents collected from different colonic sites (caecal, mucosal-associated and faecal) as depicted in Fig. 8. However, there were no statistically significant differences between the concentrations of acetate, propionate, valerate and succinate in the DSS-control and the HC mice, with the exception for butyrate levels in the caecum. Supplementation of mice with *B. coagulans*, GBRS and synbiotic induced significantly increased SCFA levels although they varied across different sample types. Overall, the highest SCFA levels were detected in caecal contents followed by faecal and mucosal-associated samples. Interestingly, *B. coagulans* alone was noted to increase the SCFA levels in the caecum while no effect was observed in SCFA levels in mucosal-associated or faecal samples except for propionate. However, synbiotic and GBRS were effective in elevating the levels of acetate, propionate and butyrate in the caecal and faecal contents compared to that of the DSS-control.

*Bacillus coagulans* (10.9 ± 0.8 μg/g), GBRS (11.2 ± 1.7 μg/g) and synbiotic (10.6 ± 1.1 μg/g) caused significant elevations in butyrate levels in the caecum compared with that of DSS-control (2.45 ± 0.4 μg/g). GBRS (11.8 ± 2.0 μg/g) and synbiotic (10.9 ± 0.7 μg/g) were also noted to increase faecal butyrate levels compared to that of DSS-control (3.72 ± 1.0 μg/g), while *B. coagulans* (6.89 ± 1.2 μg/g) had no effect. GBRS and synbiotic also increased faecal valerate levels. Synbiotic supplementation lowered succinate levels although not statistically. Such alterations in SCFAs by synbiotic supplementation indicates the excellent potential of the bioactive ingredients in inducing beneficial SCFA modulations.

## Discussion

Application of dietary strategies to prevent the onset or reduce the severity of IBD is gaining momentum. The mechanisms that contribute to managing IBD appear to act through modulating cytokine responses, epithelial integrity and gut microbiota [50, 51]. The results of the present study clearly indicated that pre-conditioning of the gut with synbiotic supplementation carrying probiotic and prebiotic components markedly reduced the symptoms and severity of DSS-induced colitis in the mouse model. The results supported the anti-inflammatory potentials of both the probiotic *B. coagulans* MTCC5856 and GBRS supplement ingredients. However, the effect was noted to be more profound with synbiotic supplementation, as illustrated by its ability to prevent the clinical manifestations, macroscopic, histological, biochemical, metabolic and immune parameter changes in the DSS-induced colitic mice. Synergistic action between the two bioactive components could account for such enhanced beneficial effect.

The feeding of DSS-induced mice with *B. coagulans*, GBRS and synbiotic supplements significantly (*p* < 0.0001) lowered the DAI scores observed by the marked reduction in body weight loss and lower incidences of diarrheic/ bleeding faeces compared to that of DSS-control mice (Fig. 2). Additionally, the supplementations prevented spleen enlargement, increase in colon weight/body weight ratio and colon shortening. These macroscopic markers are directly associated with intestinal inflammation and disease severity in experimental colitis models [35]. Green banana-supplemented diets [29] and *B. coagulans* spores [15] have each previously been shown to reduce clinical diarrheal episodes, in line with the observations on DDS-induced mice in the current study. The anti-diarrheic effect of GBRS could be due to its high RS content that, upon reaching the caecum/colon, is metabolized by bacteria to SCFAs [27]. These, in turn, stimulate salt and water absorption, provide energy and induce a trophic effect on the colon [29]. Cooking banana (*Musa acuminata*) have been previously characterised for their high RS content and prebiotic properties [52]. High RS content (38% of the total DF content) of the GBRS flour from diploid cultivars of *M. acuminata* “Lady Finger” bananas used in the current study could be accounted for the observed positive effects. The ability of *B. coagulans* to elicit the anti-diarrheic effect could be via several proposed mechanisms that include suppression and binding of pathogenic bacteria, improvement of the epithelial barrier function and alteration of the immune activity of the host [15]. Synbiotic supplementation that combines these effects should stimulate more profound efficacy outcomes against manifestations of IBD. This was supported by the observations in the current study.

Histological studies, not easily possible in human clinical trials, also showed a potential synbiotic outcome. There was a marked preservation of the structure of the epithelium of the colon of synbiotic supplemented mice (Fig. 3a, b) compared to the DSS-control, as well as to the *B. coagulans* and GBRS supplementations alone. Synbiotic supplementation also showed marked protection to the colonic epithelial architecture by alleviating crypt disruption, loss of goblet cells, submucosal oedema and inflammatory infiltrates induced by DSS.

In other biomarkers of IBD activity related to mucosal integrity, synbiotic supplementation caused a significant reduction (*p* < 0.0001) in MPO activity (Fig. 3c) especially in the DC section compared to that of DSS-control. MPO activity may cause oxidative damage to host tissue and induce or perpetuate inflammation. MPO is an important diagnostic and prognostic tool in assessing IBD status [53]. The level of MPO activity is directly proportional to the neutrophil concentration and thus is an index of neutrophil infiltration and inflammation [49]. Neutrophil-myeloperoxidase is an enzyme that catalyses the production of reactive oxygen species and is increased in the mucosa of patients with IBD [53]. This suggests that synbiotic supplementation had an anti-inflammatory effect that correlates to the histological evidence of protection.

Synbiotic supplementation in this study, followed in efficacy by *B. coagulans* and GBRS alone, were also effective in protecting the TJ proteins (ZO-1, occludin and claudin-1) in DSS-induced mice (Fig. 5). Disruption of intestinal epithelial TJs, and impaired epithelial barrier function, is a prominent event in the pathogenesis of clinical colitis that further promotes dysregulated immune reactions, thus aggravating gut inflammation. TJs maintain the epithelial barrier function by sealing the intracellular spaces between adjoining epithelial cells, consequently restricting the paracellular movement of harmful substances across intestinal mucosa [54]. Goblet cells and mucin production were also best protected by synbiotic and *B. coagulans* supplementations with GBRS alone also demonstrating a notable effect compared to that of the DSS-control (Fig. 4). A recent study [55] has demonstrated the ability of a *Bacillus* probiotic to upregulate the expression of TJ proteins in colitic mice, in line with these observations with *B. coagulans* supplementation. In our previous study, *B. coagulans* spores in synbiotic combination with sugar cane fibre showed similar beneficial effects on the epithelial barrier function in DSS-induced colitic mice [19]. While the efficacy of the synbiotic combination to stimulate TJ proteins and/or circumvent the TJ degradation by DSS needs further investigation, taken together the results support the ability of the prebiotic and probiotic combination to reinforce intestinal barrier integrity and help prevent the manifestation of IBD.

The breach of epithelial integrity in IBD also triggers aberrant inflammatory responses resulting in increased accumulation of pro-inflammatory mediators and thus further exacerbating the inflammation cascade and tissue damage [2]. *B. coagulans* alone, and in synbiotic combination, demonstrated excellent immunomodulatory and anti-inflammatory efficacy as evidenced by reduction in colonic pro-inflammatory cytokine levels of IL-1α, IL-1β, Il-6, IL-12, TNF-α, and IFN-γ in both PC and DC segments. The results were similar to our previous studies that demonstrated marked immunomodulatory effects of *B. coagulans* MTCC 5856 spores in vitro with colonic cell cultures [14] and in vivo in mice colonic tissues [19]. Levels of IL-1β, IL-6 and TNF-α were reported to be elevated in IBD patients [56]. These cytokines are mainly secreted by activated lamina propria antigen-presenting cells (APC) in response to the inflammation. APCs are part of the mechanism that maintains intestinal immune tolerance in the steady state but also prevent inappropriate responses to components of the gut microbiota that contribute to pathology in IBD [57]. TNF-α plays a pivotal role in triggering the accumulation and activation of leukocytes in colitis and hence is an important therapeutic target [58]. Blockade of IL-6 signalling with monoclonal antibodies was also reported to be effective in reducing chronic intestinal inflammation in a mouse model. This effect was associated with the activation of T cell apoptosis and the suppressed production of pro-inflammatory IFN-γ [59]. In contrast to a previous study [27] that used green dwarf banana flour and reported no effect on colonic cytokines, the current study with GBRS supplementation observed a noticeable reduction in levels of IL-1β, IL-6 and IFN-γ as well as the reduction in detected serum IL-12 levels. However, the respective immune-regulatory effects were less pronounced compared to that with synbiotic supplementation. There was also no effect on levels of colonic IL-1α, IL-12, TNF-α and serum IL-10. Furthermore, in serum, synbiotic supplementation induced a marked reduction in pro-inflammatory IL-1β levels while concomitantly increasing anti-inflammatory IL-10 indicating a synergistic effect.

*Bacillus coagulans* MTCC 5856 spores have been demonstrated to impart strong immunomodulatory effects to colonic cells in vitro in an inflammatory state [14]. This observation highlights the potential for application of probiotics with substantial immunomodulatory capacity, in conjunction with prebiotics with average immune-regulating effect, to potentiate combined anti-inflammatory effects to mitigate the aberrant immune responses in IBD. IL-10 plays a prominent role in counterbalancing Th1 and Th17 immune activity in IBD towards a Th2 response by downregulating antigen presentation and subsequent release of pro-inflammatory cytokines thereby attenuating mucosal inflammation [60]. IL-10 deficiency has been demonstrated to exacerbate colitis in the DSS-induced colitis model as IL-10^−/−^ knockout mice develop spontaneous colitis [61]. Moreover, IL-10 administration has been determined to ameliorate colitis in mice by suppressing intestinal inflammation and reducing pro-inflammatory cytokine production [62]. The anti-inflammatory potential of the synbiotic supplementation in the current study, therefore, warrants its application to human IBD trials to confirm the ability to regulate the exacerbated immune responses.

The supplementations also moderated other indicators of the inflammatory response. The *B. coagulans* and synbiotic supplementations suppressed increased colonic inducible nitric oxide synthase (iNOS) activity. Th1 and Th17 cytokines upregulate the iNOS expression and production nitric oxide (NO) in IBD that causes oxidative stress-related inflammation and tissue damage [63]. Elevated levels of CRP have been shown in human IBD [64]. When inflammation is triggered, circulating IL-6 (partly induced by IL-1β and TNF-α) stimulate the production of CRP in the liver and subsequent release into the bloodstream [65]. In the present study, the elicited levels of colonic IL-6 and serum CRP induced by DSS-induction were mitigated effectively by supplementations. Synbiotic, *B. coagulans* and GBRS supplementations displayed potent immune regulating efficacies to normalise the elevated serum CRP levels indicative of inflammation. Synbiotic was the most effective statistically (*p* < 0.0001) compared to the DSS-control (Fig. 6k). The combined immunomodulatory effect of *B. coagulans* and GBRS could, therefore, be accounted for by a potentiated synergistic efficacy of synbiotic supplementation in mitigating the pro-inflammatory cytokines. These findings indicate that the modulation of DSS-induced aberrant inflammatory responses by components of synbiotic could be due to either a direct effect via suppression of pro-inflammatory cytokine or to an indirect effect imparted by maintenance of epithelial integrity. A boost to the epithelial barrier function would result in reduction of entry of foreign luminal antigens and thus lessen full activation of the innate immune system. There are therefore, multiple mechanisms by which the action of the synbiotic combination supplementation can address the underlying mechanisms that result in IBD pathology.

The synbiotic supplementation in the current study demonstrated strong ability to modulate faecal metabolic profile of DSS-induced mice compared with that of the DSS-control (Fig. 7). *B. coagulans* and GBRS supplementations alone could not mediate the same modulations in the metabolic profiles as observed by their synbiotic combination. Microbiota-derived metabolites and SCFAs, which are the signatures of the gut microbiota and modulate immune activity in the gut, are important indicators of dysbiotic patterns in IBD [66, 67]. However, the direct characterisation of the microbiome was not possible as DNA extracted from caecal, faecal and mucosal stool samples from DSS-induced mice in the present study, failed amplification before 16 s rRNA sequencing. This was due to the presence of DSS in the samples that is known to inhibit the PCR amplification [68]. Nevertheless, the profiling of microbiota-derived untargeted metabolites revealed distinct patterns between the DSS-control and the HC samples that are in agreement with previous reports [69, 70]. There was also a significant difference in faecal metabolic profiles from IBD subjects compared to that of their healthy counterparts. A similar trend in effects was observed in terms of SCFA profiles. SCFAs made in the colon are active metabolites that function to reduce inflammatory mediators and increasing epithelial barrier function [71]. The most abundant SCFAs in the colon are acetate, propionate and butyrate. These are produced by gut microbiota via fermentation of indigestible fibres. The concentrations of SCFAs vary along the length of the gut. The caecum and proximal colon show the highest levels that then decline towards distal colon segment [72]. Moreover, in the caecum and colon 95% of SCFAs are absorbed by the colonocytes while only 5% are excreted in the faeces [73]. The synbiotic and GBRS supplementations elicited elevated SCFA production along the entire length of colon. In contrast, *B. coagulans* alone was not very effective in inducing SCFAs along the colon past the caecum. Trachsel et al. [74] observed that resistant potato starch fed to pigs was initially fermented to high concentrations of lactate, especially in caecum by various lactic acid bacteria. This lactic acid was then metabolised into butyrate by the secondary fermenters such as *Anaerostipes hadrus*, thus decreasing the level of lactate in faecal samples. The authors, therefore, projected the co-administration of resistant potato starch with the members of these bacterial groups to enhance the beneficial effects. Similarly, in our previous study [19], *B. coagulans* spores combined with whole-plant sugar cane fibre, as a synergistic supplement to DSS-colitic mice, augmented the SCFAs levels along the entire colon length. It is inferred that, beyond the caecum, fibre available for fermentation could be limited. The low DF (crude fibre + neutral detergent fibre) content in the standard rodent chow could be accounted for such effect. This conclusion is further supported by the ability of synbiotic and GBRS supplementation that showed higher levels of SCFAs production in mucosal-associated and faecal contents. GBRS treatment added 1.46 g more DF content (2 g/day/mouse) relative to unsupplemented standard chow (0.54 g/day/mice) in the GBRS and synbiotic supplemented chow diets. The similar levels of SCFA produced by both GBRS alone and synbiotic suggest there was only a limited role of the administered spore probiotic in mediating the greater SCFA levels. It is concluded that synergistically applying *B. coagulans* along with GBRS, could potentiate SCFAs production along the entire length of colon to mediate trophic beneficial effects in IBD. However, clinical trials would be needed to determine if the mouse results would also translate to the activity of the human gut microbiome.

Decreased SCFAs concentrations, particularly butyrate, have a direct effect on microbial perturbations. This results in defects in colonic barrier function and is associated with the related aberrant immune responses in IBD [18]. In vitro [75, 76] and in vivo [77] studies have determined the effectiveness of butyrate in increasing epithelial integrity and mucus secretion. In the results of this study, the considerable increase in butyrate levels by synbiotic, GBRS and *B. coagulans* supplementation could also be related to the positive effects observed on the histology of the colon, barrier integrity and reduction in disease severity in DSS-induced mice. Butyrate is the preferred energy source for colonocytes and has the ability to regulate cytokines, thus showing protection against inflammation in UC and colorectal cancer [72]. The improved expression of TJ proteins and mucus staining in goblet cells observed here could be partially attributed to the elevated butyrate levels associated with synbiotic supplementation. Metabolites such as acetate and propionate, that were found to be elevated with synbiotic supplementation along the entire colonic length, have also been found to benefit epithelial integrity via binding with metabolite-sensing G-protein-coupled receptors such as GPR43, GPR109A, and modulating immune response [78–80]. Valerate has been determined to stimulate intestinal growth and attenuate inflammatory pathogenesis in colitis [81]. This SCFA was also increased by synbiotic supplementation in this study. Therefore, a prebiotic component that directly or indirectly influences SCFA production capacity of a symbiotically administered probiotic and other beneficial gut microflora, is advantageous in modulating inflammation in IBD.

## Conclusions

The research has highlighted a substantial efficacy of a synbiotic supplementation of GBRS with *B. coagulans* spores in reducing the clinical manifestations and severity of DSS-induced colitis in a mouse model. The probiotic and prebiotic components complement each other to potentiate the beneficial effects. A substantial anti-inflammatory effect of the synbiotic supplementation was generated by suppressing aberrant immune responses and colonic damage induced by DSS. The combination of the probiotic *B. coagulans* MTCC5856 spores and GBRS also improved the production of the metabolites and SCFAs. Together these could similarly function to modulate the inflammatory parameters and ameliorate the disease severity.

The study provides valuable insights into synergistic mechanisms of synbiotic supplementation that operate by resolving dysregulated immune response, weakened mucosal barrier integrity and altered metabolic profile, thus attenuating gut inflammation. The observed synergistic functioning ameliorating or preventing the disease severity in DSS-induced mice model supports its further investigation for mitigating inflammation in human IBD. Furthermore, synergistic combinations of these synbiotic ingredients could be applied to develop novel shelf-stable foods targeted at improving gut health.

## Electronic supplementary material

Below is the link to the electronic supplementary material.

Supplementary file1 (DOCX 3164 kb)

## References

[1] Khor B, Gardet A, Xavier RJ (2011). Genetics and pathogenesis of inflammatory bowel disease. Nature.

[2] Xavier R, Podolsky D (2007). Unravelling the pathogenesis of inflammatory bowel disease. Nature.

[3] Pickard JM, Zeng MY, Caruso R, Núñez G (2017). Gut microbiota: role in pathogen colonization, immune responses, and inflammatory disease. Immunol Rev.

[4] Hou JK, Abraham B, El-Serag H (2011). Dietary intake and risk of developing inflammatory bowel disease: a systematic review of the literature. Am J Gastroenterol.

[5] Wasilewski A, Zielińska M, Storr M, Fichna J (2015). Beneficial effects of probiotics, prebiotics, synbiotics, and psychobiotics in inflammatory bowel disease. Inflamm Bowel Dis.

[6] Azad M, Kalam A, Sarker M, Li T, Yin J (2018). Probiotic species in the modulation of gut microbiota: an overview. Biomed Res Int.

[7] Currò D, Ianiro G, Pecere S, Bibbò S, Cammarota G (2017). Probiotics, fibre and herbal medicinal products for functional and inflammatory bowel disorders. Br J Pharmacol.

[8] Ghouri YA, Richards DM, Rahimi EF, Krill JT, Jelinek KA, DuPont AW (2014). Systematic review of randomized controlled trials of probiotics, prebiotics, and synbiotics in inflammatory bowel disease. Clin Exp Gastroenterol.

[9] Matijašić M, Meštrović T, Perić M, Čipčić Paljetak H, Panek M, Vranešić Bender D, Ljubas Kelečić D, Krznarić Ž, Verbanac D (2016). Modulating composition and metabolic activity of the gut microbiota in IBD patients. Int J Mol Sci.

[10] Liu H, Cui SW, Chen M, Li Y, Liang R, Xu F, Zhong F (2019). Protective approaches and mechanisms of microencapsulation to the survival of probiotic bacteria during processing, storage and gastrointestinal digestion: a review. Crit Rev Food Sci Nutr.

[11] Cutting SM (2011). *Bacillus* probiotics. Food Microbiol.

[12] Elshaghabee FMF, Rokana N, Gulhane RD, Sharma C, Panwar H (2017). *Bacillus* as potential probiotics: status, concerns, and future perspectives. Front Microbiol.

[13] Majeed M, Majeed S, Nagabhushanam K, Natarajan S, Sivakumar A, Ali F (2016). Evaluation of the stability of *Bacillus coagulans* MTCC 5856 during processing and storage of functional foods. Int J Food Sci Technol.

[14] Shinde T, Vemuri R, Shastri MD, Perera AP, Tristram S, Stanley R, Eri R (2019). Probiotic *Bacillus coagulans* MTCC 5856 spores exhibit excellent in vitro functional efficacy in simulated gastric survival, mucosal adhesion and immunomodulation. J Funct Foods.

[15] Majeed M, Nagabhushanam K, Natarajan S, Sivakumar A, Ali F, Pande A, Majeed S, Karri SK (2016). *Bacillus coagulans* MTCC 5856 supplementation in the management of diarrhea predominant Irritable Bowel syndrome: a double blind randomized placebo controlled pilot clinical study. Nutr J.

[16] Dobson A, Cotter PD, Ross RP, Hill C (2012). Bacteriocin production: a probiotic trait?. Appl Environ Microbiol.

[17] Gaspar C, Donders G, Palmeira-de-Oliveira R, Queiroz J, Tomaz C, Martinez-de-Oliveira J, Palmeira-de-Oliveira A (2018). Bacteriocin production of the probiotic *Lactobacillus acidophilus* KS400. AMB Express.

[18] van der Beek CM, Dejong CH, Troost FJ, Masclee AA, Lenaerts K (2017). Role of short-chain fatty acids in colonic inflammation, carcinogenesis, and mucosal protection and healing. Nutr Rev.

[19] Shinde T, Perera AP, Vemuri R, Gondalia SV, Karpe AV, Beale DJ, Shastri S, Southam B, Eri R, Stanley R (2019). Synbiotic supplementation containing whole plant sugar cane fibre and probiotic spores potentiates protective synergistic effects in mouse model of IBD. Nutrients.

[20] Kolida S, Gibson GR (2011). Synbiotics in health and disease. Annu Rev Food Sci Technol.

[21] Walton SL, Bischoff KM, van Heiningen AR, van Walsum GP (2010). Production of lactic acid from hemicellulose extracts by *Bacillus coagulans* MXL-9. J Ind Microbiol Biotechnol.

[22] Majeed M, Nagabhushanam K, Arumugam S, Natarajan S, Majeed S, Pande A, Beede K, Ali F (2018). Cranberry seed fibre: a promising prebiotic fibre and its fermentation by the probiotic *Bacillus coagulans* MTCC 5856. Int J Food Sci Technol.

[23] Majeed M, Majeed S, Nagabhushanam K, Arumugam S, Natarajan S, Beede K, Ali F (2018). Galactomannan from *Trigonella foenum‐graecum* L. seed: prebiotic application and its fermentation by the probiotic *Bacillus coagulans* strain MTCC 5856. Food Sci Nutr.

[24] Nugent AP (2005). Health properties of resistant starch. Nutr Bull.

[25] Lockyer S, Nugent A (2017). Health effects of resistant starch. Nutr Bull.

[26] Scarminio V, Fruet AC, Witaicenis A, Rall VL, Di Stasi LC (2012). Dietary intervention with green dwarf banana flour (*Musa* sp. AAA) prevents intestinal inflammation in a trinitrobenzenesulfonic acid model of rat colitis. Nutr Res.

[27] Almeida-Junior L, Curimbaba T, Chagas A, Quaglio A, Di Stasi L (2017). Dietary intervention with green dwarf banana flour (*Musa* sp. AAA) modulates oxidative stress and colonic SCFAs production in the TNBS model of intestinal inflammation. J Funct Foods.

[28] Rabbani G, Larson C, Islam R, Saha U, Kabir A (2010). Green banana-supplemented diet in the home management of acute and prolonged diarrhoea in children: a community-based trial in rural Bangladesh. Trop Med Int Health.

[29] Rabbani GH, Teka T, Zaman B, Majid N, Khatun M, Fuchs GJ (2001). Clinical studies in persistent diarrhea: dietary management with green banana or pectin in Bangladeshi children. Gastroenterology.

[30] dos Santos Alves LAA, Lorenzo JM, Gonçalves CAA, dos Santos BA, Heck RT, Cichoski AJ, Campagnol PCB (2016). Production of healthier bologna type sausages using pork skin and green banana flour as a fat replacers. Meat Sci.

[31] Yangılar F (2015). Effects of green banana flour on the physical, chemical and sensory properties of ice cream. Food Technol Biotechnol.

[32] Vogado CdO, Leandro EdS, Zandonadi RP, de Alencar ER, Ginani VC, Nakano EY, Habú S, Aguiar PA (2018). Enrichment of probiotic fermented milk with green banana pulp: characterization microbiological. Physicochem Sens Nutr.

[33] Demon D, Kuchmiy A, Fossoul A, Zhu Q, Kanneganti T-D, Lamkanfi M (2014). Caspase-11 is expressed in the colonic mucosa and protects against dextran sodium sulfate-induced colitis. Mucosal Immunol.

[34] Li P, Zhang R, Wang L, Gan Y, Xu Y, Song L, Luo L, Zhao C, Zhang C, Ouyang B (2017). Long-term load duration induces N-cadherin down-regulation and loss of cell phenotype of nucleus pulposus cells in a disc bioreactor culture. Biosci Rep.

[35] Chassaing B, Aitken JD, Malleshappa M, Vijay‐Kumar M (2014). Dextran sulfate sodium (DSS)‐induced colitis in mice. Cur Protoc Immunol.

[36] Perera AP, Fernando R, Shinde T, Gundamaraju R, Southam B, Sohal SS, Robertson AA, Schroder K, Kunde D, Eri R (2018). MCC950, a specific small molecule inhibitor of NLRP3 inflammasome attenuates colonic inflammation in spontaneous colitis mice. Sci Rep.

[37] Mei Q, Xu J, Xiang L, Hu Y, Hu X, Xu Z (2005). Change of nitric oxide in experimental colitis and its inhibition by melatonin in vivo and in vitro. Postgrad Med J.

[38] Vemuri R, Shinde T, Gundamaraju R, Gondalia S, Karpe A, Beale D, Martoni C, Eri R (2018). *Lactobacillus acidophilus* DDS-1 modulates the gut microbiota and improves metabolic profiles in aging mice. Nutrients.

[39] Furuhashi T, Sugitate K, Nakai T, Jikumaru Y, Ishihara G (2018). Rapid profiling method for mammalian feces short chain fatty acids by GC-MS. Anal Biochem.

[40] Sansone S-A, Fan T, Goodacre R, Griffin JL, Hardy NW, Kaddurah-Daouk R, Kristal BS, Lindon J, Mendes P, Morrison N (2007). The metabolomics standards initiative. Nat Biotechnol.

[41] Smart KF, Aggio RB, Van Houtte JR, Villas-Bôas SG (2010). Analytical platform for metabolome analysis of microbial cells using methyl chloroformate derivatization followed by gas chromatography–mass spectrometry. Nat Protoc.

[42] Beale DJ, Marney D, Marlow DR, Morrison PD, Dunn MS, Key C, Palombo EA (2013). Metabolomic analysis of *Cryptosporidium parvum* oocysts in water: a proof of concept demonstration. Environ Pollut.

[43] Karpe AV, Beale DJ, Harding IH, Palombo EA (2015). Optimization of degradation of winery-derived biomass waste by Ascomycetes. J Chem Technol Biotechnol.

[44] Beale D, Morrison P, Key C, Palombo E (2014). Metabolic profiling of biofilm bacteria known to cause microbial influenced corrosion. Water Sci Technol.

[45] French KE, Harvey J, McCullagh JS (2018). Targeted and untargeted metabolic profiling of wild grassland plants identifies antibiotic and anthelmintic compounds targeting pathogen physiology, metabolism and reproduction. Sci Rep.

[46] Sun H, Zhang A, Yan G, Piao C, Li W, Sun C, Wu X, Li X, Chen Y, Wang X (2013). Metabolomic analysis of key regulatory metabolites in hepatitis C virus-infected tree shrews. Mol Cell Proteom.

[47] Hayakawa K, Matsuda F, Shimizu H (2016). Metabolome analysis of *Saccharomyces cerevisiae* and optimization of culture medium for S-adenosyl-l-methionine production. AMB Express.

[48] Srutkova D, Schwarzer M, Hudcovic T, Zakostelska Z, Drab V, Spanova A, Rittich B, Kozakova H, Schabussova I (2015). *Bifidobacterium longum* CCM 7952 promotes epithelial barrier function and prevents acute DSS-induced colitis in strictly strain-specific manner. PLoS One.

[49] Han F, Fan H, Yao M, Yang S, Han J (2017). Oral administration of yeast β-glucan ameliorates inflammation and intestinal barrier in dextran sodium sulfate-induced acute colitis. J Funct Foods.

[50] Al Mijan M, Lim BO (2018). Diets, functional foods, and nutraceuticals as alternative therapies for inflammatory bowel disease: Present status and future trends. World J Gastroenterol.

[51] Ferguson LR, Shelling AN, Browning BL, Huebner C, Petermann I (2007). Genes, diet and inflammatory bowel disease. Mutat Res Fundam Mol Mech Mutagen.

[52] Lehmann U, Jacobasch G, Schmiedl D (2002). Characterization of resistant starch type III from banana (*Musa acuminata*). J Agric Food Chem.

[53] Hansberry DR, Shah K, Agarwal P, Agarwal N (2017). Fecal myeloperoxidase as a biomarker for inflammatory bowel disease. Cureus.

[54] Turner JR (2009). Intestinal mucosal barrier function in health and disease. Nat Rev Immunol.

[55] Gong Y, Li H, Li Y (2016). Effects of *Bacillus subtilis* on epithelial tight junctions of mice with inflammatory bowel disease. J Interferon Cytokine Res.

[56] Neurath MF (2014). Cytokines in inflammatory bowel disease. Nat Rev Immunol.

[57] Mann ER, Li X (2014). Intestinal antigen-presenting cells in mucosal immune homeostasis: crosstalk between dendritic cells, macrophages and B-cells. World J Gastroenterol.

[58] Hyams JS, Lerer T, Griffiths A, Pfefferkorn M, Kugathasan S, Evans J, Otley A, Carvalho R, Mack D, Bousvaros A (2008). Long-term outcome of maintenance infliximab therapy in children with Crohn's disease. Inflamm Bowel Dis.

[59] Atreya R, Mudter J, Finotto S, Müllberg J, Jostock T, Wirtz S, Schütz M, Bartsch B, Holtmann M, Becker C (2000). Blockade of interleukin 6 trans signaling suppresses T-cell resistance against apoptosis in chronic intestinal inflammation: evidence in crohn disease and experimental colitis in vivo. Nat Med.

[60] Neuman MG (2007). Immune dysfunction in inflammatory bowel disease. Transl Res.

[61] Mizoguchi A, Mizoguchi E, Takedatsu H, Blumberg RS, Bhan AK (2002). Chronic intestinal inflammatory condition generates IL-10-producing regulatory B cell subset characterized by CD1d upregulation. Immunity.

[62] Tomoyose M, Mitsuyama K, Ishida H, Toyonaga A, Tanikawa K (1998). Role of interleukin-10 in a murine model of dextran sulfate sodium-induced colitis. Scand J Gastroenterol.

[63] Soufli I, Toumi R, Rafa H, Touil-Boukoffa C, Huber S (2016). Cytokines and nitric oxide in immunopathogenesis of IBD and potential therapeutic approaches. New insights into inflammatory bowel disease.

[64] Solem CA, Loftus EV, Tremaine WJ, Harmsen WS, Zinsmeister AR, Sandborn WJ (2005). Correlation of C-reactive protein with clinical, endoscopic, histologic, and radiographic activity in inflammatory bowel disease. Inflamm Bowel Dis.

[65] Del Giudice M, Gangestad SW (2018). Rethinking IL-6 and CRP: Why they are more than inflammatory biomarkers, and why it matters. Brain Behav Immun.

[66] Levy M, Thaiss CA, Elinav E (2016). Metabolites: messengers between the microbiota and the immune system. Genes Dev.

[67] Vernocchi P, Del Chierico F, Putignani L (2016). Gut microbiota profiling: metabolomics based approach to unravel compounds affecting human health. Front Microbiol.

[68] Kerr T, Ciorba M, Matsumoto H, Davis V, Luo J, Kennedy S, Xie Y, Shaker A, Dieckgraefe B, Davidson N (2011). Dextran sodium sulfate inhibition of real-time polymerase chain reaction amplification: a poly-A purification solution. Inflamm Bowel Dis.

[69] Martin F-P, Su M-M, Xie G-X, Guiraud SP, Kussmann M, Godin J-P, Jia W, Nydegger A (2017). Urinary metabolic insights into host-gut microbial interactions in healthy and IBD children. World J Gastroenterol.

[70] Marchesi JR, Holmes E, Khan F, Kochhar S, Scanlan P, Shanahan F, Wilson ID, Wang Y (2007). Rapid and noninvasive metabonomic characterization of inflammatory bowel disease. J Proteome Res.

[71] Fernández J, Redondo-Blanco S, Gutierrez-del-Rio I, Miguélez EM, Villar CJ, Lombo F (2016). Colon microbiota fermentation of dietary prebiotics towards short-chain fatty acids and their roles as anti-inflammatory and antitumour agents: a review. J Funct Foods.

[72] Koh A, De Vadder F, Kovatcheva-Datchary P, Bäckhed F (2016). From dietary fiber to host physiology: short-chain fatty acids as key bacterial metabolites. Cell.

[73] den Besten G, van Eunen K, Groen AK, Venema K, Reijngoud D-J, Bakker BM (2013). The role of short-chain fatty acids in the interplay between diet, gut microbiota, and host energy metabolism. J Lipid Res.

[74] Trachsel J, Briggs C, Gabler NK, Allen HK, Loving CL (2019). Dietary resistant potato starch alters intestinal microbial communities and their metabolites, and markers of immune regulation and barrier function in swine. Front Immunol.

[75] Nielsen DSG, Jensen BB, Theil PK, Nielsen TS, Knudsen KEB, Purup S (2018). Effect of butyrate and fermentation products on epithelial integrity in a mucus-secreting human colon cell line. J Funct Foods.

[76] Zheng L, Kelly CJ, Battista KD, Schaefer R, Lanis JM, Alexeev EE, Wang RX, Onyiah JC, Kominsky DJ, Colgan SP (2017). Microbial-derived butyrate promotes epithelial barrier function through IL-10 receptor-dependent repression of claudin-2. J Immunol.

[77] Simeoli R, Mattace Raso G, Pirozzi C, Lama A, Santoro A, Russo R, Montero-Melendez T, Berni Canani R, Calignano A, Perretti M (2017). An orally administered butyrate-releasing derivative reduces neutrophil recruitment and inflammation in dextran sulphate sodium-induced murine colitis. Br J Pharmacol.

[78] Maslowski KM, Vieira AT, Ng A, Kranich J, Sierro F, Di Y, Schilter HC, Rolph MS, Mackay F, Artis D, Xavier RJ, Teixeira MM, Mackay CR (2009) Regulation of inflammatory responses by gut microbiota and chemoattractant receptor GPR43. Nature 461:1282. 10.1038/nature08530. https://www.nature.com/articles/nature08530#supplementary-information PMC3256734 19865172

[79] Tedelind S, Westberg F, Kjerrulf M, Vidal A (2007). Anti-inflammatory properties of the short-chain fatty acids acetate and propionate: a study with relevance to inflammatory bowel disease. World J Gastroenterol.

[80] Macia L, Tan J, Vieira AT, Leach K, Stanley D, Luong S, Maruya M, Ian McKenzie C, Hijikata A, Wong C, Binge L, Thorburn AN, Chevalier N, Ang C, Marino E, Robert R, Offermanns S, Teixeira MM, Moore RJ, Flavell RA, Fagarasan S, Mackay CR (2015) Metabolite-sensing receptors GPR43 and GPR109A facilitate dietary fibre-induced gut homeostasis through regulation of the inflammasome. Nat Commun 6:6734. 10.1038/ncomms7734. https://www.nature.com/articles/ncomms7734#supplementary-information 25828455

[81] Yuille S, Reichardt N, Panda S, Dunbar H, Mulder IE (2018). Human gut bacteria as potent class I histone deacetylase inhibitors in vitro through production of butyric acid and valeric acid. PLoS One.

