# Supplementary material for: Synbiotic supplementation with prebiotic green banana resistant starch and probiotic Bacillus coagulans spores ameliorates gut inflammation in mouse model of inflammatory bowel diseases
Source: Eur J Nutr. 2020 Feb 17;59(8):3669–89. doi: 10.1007/s00394-020-02200-9 (PMC7669818; doi:10.1007/s00394-020-02200-9)
Supplement: Supplementary file 1 — Supplementary file1 (DOCX 3164 kb) [file 394_2020_2200_MOESM1_ESM.docx]

**Synbiotic supplementation with prebiotic green banana resistant starch and probiotic *Bacillus* *coagulans* spores ameliorates gut inflammation in mouse model of IBD**

**Authors:** Tanvi Shinde^1,2*^ , Agampodi Promoda Perera^2^, Ravichandra Vemuri^2^, Shakuntla V. Gondalia^3,4^, David J. Beale^5^, Avinash V. Karpe^5^, Sonia Shastri^2^, Waheedha Basheer^2^, Benjamin Southam^2^, Rajaraman Eri^2^, Roger Stanley^1*^

^1^Centre for Food Safety and Innovation, Tasmanian Institute of Agriculture, University of Tasmania, Launceston, Tasmania 7250 Australia.

^2^School of Health Sciences, College of Health and Medicine, University of Tasmania, Launceston, Tasmania 7250 Australia.

^3^Centre for Human Psychopharmacology, Swinburne University of Technology, Hawthorn, Victoria, Australia.

^4^ Health and Biosecurity, Commonwealth Scientific and Industrial Research Organization (CSIRO), Gate 13 Kintore Avenue 5000, South Australia, Australia

^5^Land and Water, Commonwealth Scientific and Industrial Research Organization (CSIRO), Ecosciences Precinct, Dutton Park 4102, Queensland, Australia.

***Correspondence**: Tanvi Shinde

Email: tanvi.shinde@utas.edu.au

Phone: +61 4 79107345

Roger Stanley

Email: roger.stanley@utas.edu.au

Phone: +61 4 34565167

**Keywords:** Synbiotic, Prebiotic, Probiotic, IBD, *Bacillus* spores, green banana, resistant starch, mucosal barrier, SCFAs

**Supplementary data**

**Supplementary Table 1.** **Nutritional information of Green Banana Resistant Starch flour**

| **Nutrient information of Natural Evolution™ Green Banana Resistant Starch flour^a^**  **(quantity per 100gm)** |
| --- |
| Energy 1412 kj  Protein 3.2 g  Total fat < 1 g  Saturated < 1 g  Monounsaturated < 1 g  Polyunsaturated < 1 g  Trans < 1 g  Dietary fibre (total) 50 g  Resistant 38 g  Carbohydrates available 37 g  Total sugar (sucrose) < 1 g  Sodium < 1 g  Potassium 1230 mg  Zinc 6.2 mg  Magnesium 121 mg  Vitamin E 1.8 IU  Manganese 3.7 mg  Inulin 4 g  Vitamin B3 530 μg  Vitamin B6 260 μg  5HTP (5 Hydroxytryptophan) 13.2 mg |
| ^a^Ingredients: *Musa acuminata* cv Lady Finger, Qld, Australia. Information sourced from [www.naturalevolutionfoods.com.au](http://www.naturalevolutionfoods.com.au) |

**Supplementary Table 2.** **Nutritional information of mice standard chow diet**

| **Nutrient information of standard rodent chow diet - Barastoc Mice Cubes^a^** | |
| --- | --- |
| Minimal crude protein 20 %  Minimal crude fat 6 %  Crude fibre 3.2 %  Acid detergent fibre 4.4 %  Neutral detergent fibre 10.4 %  Digestible energy 12.8 MJ/kg  Calcium 1.14 %  Phosphorus 0.94 %  Sodium 0.35 %  Potassium 0.82 %  Chloride 0.58 %  Magnesium 0.24 %  Lysine 1.11 %  Methionine 0.37 %  Linoleic 1.52 %  Starch 29 %  Vitamin A 15 IU/g  Vitamin D3 2 IU/g  Vitamin E 260 mg/kg  Vitamin K3 55 mg/kg | Vitamin A 15 IU/g  Vitamin D3 2 IU/g  Vitamin E 260 mg/kg  Vitamin K3 55 mg/kg  Vitamin B1 64 mg/kg  Vitamin B2 48 mg/kg  Vitamin B6 30 mg/kg  Vitamin B12 0.08 mg/kg  Niacin 400 mg/kg  Panto 220 mg/kg  Biotin 1.48 mg/kg  Folic 11 mg/kg  Iron 51 mg/kg  Zinc 60 mg/kg  Manganese 120 mg/kg  Copper 10 mg/kg  Selenium 0.1 mg/kg  Molybdenum 0.4 mg/kg  Cobalt 0.6 mg/kg  Iodine 1 .4 mg/kg |
| ^a^ Ingredients include: wheat, wheat byproducts, groats (dehulled oats), meat meal, canola oil, soyabean meal, skim milk powder, molasses, salt, vitamins, trace minerals. The product is a complete and balanced diet to support the growth and health of mice and rats in laboratory environment.  Information sourced from [www.ridley.com.au](http://www.ridley.com.au) Product code 102108 | |

**Supplementary Fig. 1 Non-significant effect of *B. coagulans* spores, GBRS and synbiotic on immune markers in colon tissues and blood serum.** Protein levels of cytokines including (**A**) IL-10 (**B**) IL-17, (**C**) MIP-1α, (**D**) MIP-1β, (**E**) GM-CSF in proximal and distal colon explants as well as cytokine levels of (**F**) IL-1α, (**G**) IL-6, (**H**) TNF-α, (**I**) IL-17, (**J**) IFN-γ, (**K**) MIP-1α, (**L**) MIP-1β, (**M**) GM-CSF in blood serum were analysed by Bio-plex. Statistical significance among groups evaluated by one-way ANOVA followed by Tukey’s test. Non-significant (ns) vs. DSS-colitic group and data expressed as mean ± SEM (*n* = 3 per group).

**A.**

**B.**

**C.**

**D.**

**F.**

**E.**

**G.**

**H.**

**I.**

**J.**

**K.**

**L.**

**M.**

**Supplementary Table 3. Most significant compounds identified by OPLS-DA and SAM analysis in HC, DSS-control, *B. coagulans* (BC), GBRS and Synbiotic groups** (*****First 28 compounds are identified by SAM).

| **Compound name** | **InCHI Key** | **DSS-control**  **(FC)** | **BC**  **(FC)** | **HC**  **(FC)** | **GBRS**  **(FC)** | **Syn-biotic**  **(FC)** | **SAM (*P* value)** |
| --- | --- | --- | --- | --- | --- | --- | --- |
| Uracil* | ISAKRJDGNUQOIC-UHFFFAOYSA-N | 1.0273 | 0.93785 | 1.3456 | 0.92366 | 1.0277 | 0.0032787 |
| Lyxosylamine* | RQBSUMJKSOSGJJ-AGQMPKSLSA-N | 2.0177 | 1.8516 | 2.0713 | 1.9025 | 1.7637 | 0.004918 |
| Linoleic acid* | OYHQOLUKZRVURQ-HZJYTTRNSA-N | 0.91878 | 0.79878 | 1.0371 | 0.82761 | 0.77441 | 0.0059016 |
| Glycerol* | PEDCQBHIVMGVHV-UHFFFAOYSA-N | 1.1377 | 0.98485 | 0.20374 | 1.0278 | 1.0753 | 0.0062295 |
| Hypoxanthine* | FDGQSTZJBFJUBT-UHFFFAOYSA-N | 1.0261 | 0.90697 | 1.2559 | 0.92141 | 0.96043 | 0.0063934 |
| Threonine* | AYFVYJQAPQTCCC-GBXIJSLDSA-N | 1.1089 | 1.024 | 1.4418 | 1.0043 | 0.98399 | 0.0080328 |
| Nicotinic acid* | PVNIIMVLHYAWGP-UHFFFAOYSA-N | 0.58642 | 0.55666 | 0.78919 | 0.57325 | 0.54117 | 0.017213 |
| Stearic acid* | QIQXTHQIDYTFRH-UHFFFAOYSA-N | 1.1904 | 1.1258 | 0.089547 | 1.1692 | 1.1284 | 0.021967 |
| Palmitic acid* | IPCSVZSSVZVIGE-UHFFFAOYSA-N | 0.97012 | 0.97602 | 1.2616 | 1.0181 | 0.97063 | 0.022131 |
| L-alanine* | MUBZPKHOEPUJKR-UHFFFAOYSA-N | 1.4478 | 1.3039 | 1.2798 | 1.359 | 1.2537 | 0.024098 |
| Glycine* | DHMQDGOQFOQNFH-UHFFFAOYSA-N | 0.74802 | 0.82314 | 0.96068 | 0.83011 | 0.80656 | 0.030328 |
| Myristic acid* | TUNFSRHWOTWDNC-UHFFFAOYSA-N | 0.93638 | 0.98458 | 1.2561 | 1.0268 | 0.93831 | 0.038033 |
| Oleic acid* | ZQPPMHVWECSIRJ-KTKRTIGZSA-N | 0.90587 | 0.82736 | 1.1791 | 0.81412 | 0.81932 | 0.040656 |
| Tagatose* | LKDRXBCSQODPBY-OEXCPVAWSA-N | 0.76788 | 1.2614 | 1.0012 | 1.316 | 1.2004 | 0.042459 |
| Altrose* | WQZGKKKJIJFFOK-VSOAQEOCSA-N | 0.19196 | 0.81434 | 0.24376 | 0.82613 | 0.8301 | 0.060164 |
| Glucose* | WQZGKKKJIJFFOK-GASJEMHNSA-N | 0.76446 | 0.8646 | 0.54832 | 0.85449 | 1.0548 | 0.068689 |
| Urea* | XSQUKJJJFZCRTK-UHFFFAOYSA-N | 1.8628 | 2.1847 | 2.4975 | 2.2657 | 2.0866 | 0.072787 |
| Talose* | WQZGKKKJIJFFOK-WHZQZERISA-N | 0.32594 | 0.6913 | 0.39272 | 0.71034 | 0.77841 | 0.077869 |
| Oxalic acid* | KZSNJWFQEVHDMF-BYPYZUCNSA-N | 1.5115 | 1.326 | 2.0337 | 1.3618 | 1.2644 | 0.095246 |
| Cellobiose2* | DLRVVLDZNNYCBX-ABXHMFFYSA-N | 0.53979 | 0.7158 | 0.24935 | 0.48633 | 0.95198 | 0.096721 |
| Cholic acid* | BHQCQFFYRZLCQQ-OELDTZBJSA-N | 0.86044 | 0.76698 | 1.105 | 0.78972 | 0.75477 | 0.10689 |
| Allo-inositol* | CDAISMWEOUEBRE-UHFFFAOYSA-N | 0.35389 | 0.36113 | 0.47427 | 0.35022 | 0.40925 | 0.11574 |
| Cellobiose1* | DLRVVLDZNNYCBX-ABXHMFFYSA-N | 0.51337 | 0.66024 | 0.29171 | 0.461 | 0.95338 | 0.11574 |
| Tyrosine* | OUYCCCASQSFEME-QMMMGPOBSA-N | 0.33892 | 0.4797 | 0.34097 | 0.41096 | 0.68526 | 0.1359 |
| 4-Guanidinobutyric acid* | TUHVEAJXIMEOSA-UHFFFAOYSA-N | 1.18 | 1.0594 | 0.89388 | 1.1051 | 1.0098 | 0.15246 |
| Melibiose* | DLRVVLDZNNYCBX-ABXHMFFYSA-N | 0.5164 | 0.65741 | 0.33806 | 0.47147 | 0.97777 | 0.16754 |
| Lactose* | GUBGYTABKSRVRQ-DCSYEGIMSA-N | 0.58297 | 0.71169 | 0.31237 | 0.52576 | 0.98415 | 0.17377 |
| Allose* | WQZGKKKJIJFFOK-IVMDWMLBSA-N | 0.53556 | 0.81397 | 0.66347 | 0.84108 | 0.79865 | 0.17557 |
| Name (unidentified) | - | 1.5577 | 1.3491 | 0.049086 | 1.4048 | 1.2883 |  |
| L-lactic acid | JVTAAEKCZFNVCJ-REOHCLBHSA-N | 1.7802 | 1.5843 | 0.58119 | 1.633 | 1.5381 |  |
| Glycolic acid | AEMRFAOFKBGASW-UHFFFAOYSA-N | 1.1274 | 0.99067 | 0.029675 | 1.0337 | 0.94309 |  |
| L-valine | KZSNJWFQEVHDMF-BYPYZUCNSA-N | 0.678 | 0.63382 | 0.61791 | 0.64103 | 0.64384 |  |
| Benzoic acid | WPYMKLBDIGXBTP-UHFFFAOYSA-N | 0.88099 | 0.83002 | 0.70659 | 0.86558 | 0.79141 |  |
| L-norleucine | LRQKBLKVPFOOQJ-YFKPBYRVSA-N | 1.0204 | 0.92566 | 1.107 | 0.94936 | 0.90537 |  |
| Phosphoric acid | NBIIXXVUZAFLBC-UHFFFAOYSA-N | 0.80246 | 0.94969 | 1.0798 | 0.77205 | 0.93011 |  |
| DL-isoleucine | AGPKZVBTJJNPAG-UHFFFAOYSA-N | 0.6982 | 0.60584 | 0.73781 | 0.62697 | 0.59691 |  |
| L-proline | ONIBWKKTOPOVIA-BYPYZUCNSA-N | 1.0582 | 0.93442 | 0.50926 | 0.95271 | 0.9057 |  |
| Succinic acid | KDYFGRWQOYBRFD-UHFFFAOYSA-N | 1.4641 | 1.4847 | 1.5977 | 1.3243 | 1.4193 |  |
| Glyceric acid | RBNPOMFGQQGHHO-UWTATZPHSA-N | 1.2087 | 1.1497 | 0.30975 | 1.1809 | 1.1151 |  |
| L-serine | MTCFGRXMJLQNBG-REOHCLBHSA-N | 1.1618 | 1.0619 | 0.3067 | 1.072 | 1.0112 |  |
| Thymine | RWQNBRDOKXIBIV-UHFFFAOYSA-N | 0.91938 | 1.0125 | 1.1993 | 0.9628 | 0.9986 |  |
| Malonic acid | OFOBLEOULBTSOW-UHFFFAOYSA-N | 0.17821 | 0.2255 | 0.13648 | 0.18249 | 0.21455 |  |
| Iminodiacetic acid | NBZBKCUXIYYUSX-UHFFFAOYSA-N | 0.35767 | 0.39998 | 0.2326 | 0.38002 | 0.44316 |  |
| Methionine | FFEARJCKVFRZRR-BYPYZUCNSA-N | 0.22457 | 0.21064 | 0.22809 | 0.21886 | 0.30851 |  |
| Aspartic acid | CKLJMWTZIZZHCS-REOHCLBHSA-N | 1.2721 | 1.1628 | 1.3098 | 1.1606 | 1.1147 |  |
| Phenylethylamine | BHHGXPLMPWCGHP-UHFFFAOYSA-N | 0.59816 | 0.90911 | 0.41977 | 0.87604 | 0.92222 |  |
| Alpha ketoglutaric acid | KPGXRSRHYNQIFN-UHFFFAOYSA-N | 0.67612 | 0.59347 | 0.36947 | 0.60824 | 0.56495 |  |
| Glutamic acid | WHUUTDBJXJRKMK-VKHMYHEASA-N | 0.81057 | 0.82398 | 0.53876 | 0.74065 | 0.94431 |  |
| 5-Aminovaleric acid | JJMDCOVWQOJGCB-UHFFFAOYSA-N | 0.62455 | 0.843 | 0.12578 | 0.77829 | 0.84164 |  |
| Lyxose | SRBFZHDQGSBBOR-AGQMPKSLSA-N | 0.47873 | 0.86784 | 0.61277 | 0.74513 | 0.82574 |  |
| Threitol | UNXHWFMMPAWVPI-QWWZWVQMSA-N | 1.5245 | 1.3159 | 1.7783 | 1.371 | 1.2521 |  |
| Arabitol | HEBKCHPVOIAQTA-QWWZWVQMSA-N | 1.5245 | 1.3159 | 1.7783 | 1.371 | 1.2521 |  |
| 6-Deoxy-D-glucose | SHZGCJCMOBCMKK-GASJEMHNSA-N | 1.1433 | 1.0515 | 1.2371 | 1.0452 | 1.0384 |  |
| Xylitol | HEBKCHPVOIAQTA-NGQZWQHPSA-N | 0.76823 | 0.74181 | 0.88974 | 0.69986 | 0.71695 |  |
| Sorbose | LKDRXBCSQODPBY-AMVSKUEXSA-N | 0.63081 | 1.0433 | 0.84948 | 1.0828 | 0.99622 |  |
| Allantoin | POJWUDADGALRAB-UHFFFAOYSA-N | 1.6539 | 1.6207 | 1.9278 | 1.6881 | 1.5712 |  |
| Galactose | WQZGKKKJIJFFOK-SVZMEOIVSA-N | 0.53147 | 0.80746 | 0.68121 | 0.83323 | 0.79426 |  |
| Tyramine | DZGWFCGJZKJUFP-UHFFFAOYSA-N | 0.56357 | 0.58491 | 0.60375 | 0.52915 | 0.57178 |  |
| Lysine | KDXKERNSBIXSRK-YFKPBYRVSA-N | 0.68847 | 0.6576 | 0.87344 | 0.65001 | 0.62596 |  |
| Sucrose | CZMRCDWAGMRECN-UGDNZRGBSA-N | 0.71034 | 0.90616 | 0.89953 | 0.71426 | 0.91998 |  |
| Cholesterol | HVYWMOMLDIMFJA-DPAQBDIFSA-N | 1.0646 | 0.96385 | 0.63693 | 0.96607 | 0.99383 |  |

(International Chemical Identifiers (InChI) and standard InChI hashes (InChIKey); FC = Fold change)
